# Map-based cloning and functional characterization reveal *CDF3* as the causal gene for the flowering time phenotype in *Brassica rapa* and *Brassica napus*

**DOI:** 10.1093/hr/uhaf324

**Published:** 2025-11-27

**Authors:** Qianru Ma, Zhi Zhao, Kede Liu, Huaxin Li, Youjuan Quan, Long Wang, Hongping Zhao, Damei Pei, Guoyong Tang, Liang Xu, Lu Xiao, Dezhi Du

**Affiliations:** Academy of Agricultural and Forestry Sciences, Qinghai University, Xining 810016, China; Key Laboratory of Spring Rapeseed Genetic Improvement of Qinghai Province, Laboratory for Research and Utilization of Qinghai Tibet Plateau Germplasm Resources, National Key Laboratory Breeding Base for Innovation and Utilization of Plateau Crop Germplasm, Xining 810016, China; Academy of Agricultural and Forestry Sciences, Qinghai University, Xining 810016, China; Key Laboratory of Spring Rapeseed Genetic Improvement of Qinghai Province, Laboratory for Research and Utilization of Qinghai Tibet Plateau Germplasm Resources, National Key Laboratory Breeding Base for Innovation and Utilization of Plateau Crop Germplasm, Xining 810016, China; National Key Laboratory of Crop Genetic Improvement, Huazhong Agricultural University, Wuhan, Hubei 430070, China; Academy of Agricultural and Forestry Sciences, Qinghai University, Xining 810016, China; Key Laboratory of Spring Rapeseed Genetic Improvement of Qinghai Province, Laboratory for Research and Utilization of Qinghai Tibet Plateau Germplasm Resources, National Key Laboratory Breeding Base for Innovation and Utilization of Plateau Crop Germplasm, Xining 810016, China; Academy of Agricultural and Forestry Sciences, Qinghai University, Xining 810016, China; Key Laboratory of Spring Rapeseed Genetic Improvement of Qinghai Province, Laboratory for Research and Utilization of Qinghai Tibet Plateau Germplasm Resources, National Key Laboratory Breeding Base for Innovation and Utilization of Plateau Crop Germplasm, Xining 810016, China; Academy of Agricultural and Forestry Sciences, Qinghai University, Xining 810016, China; Key Laboratory of Spring Rapeseed Genetic Improvement of Qinghai Province, Laboratory for Research and Utilization of Qinghai Tibet Plateau Germplasm Resources, National Key Laboratory Breeding Base for Innovation and Utilization of Plateau Crop Germplasm, Xining 810016, China; Academy of Agricultural and Forestry Sciences, Qinghai University, Xining 810016, China; Key Laboratory of Spring Rapeseed Genetic Improvement of Qinghai Province, Laboratory for Research and Utilization of Qinghai Tibet Plateau Germplasm Resources, National Key Laboratory Breeding Base for Innovation and Utilization of Plateau Crop Germplasm, Xining 810016, China; Academy of Agricultural and Forestry Sciences, Qinghai University, Xining 810016, China; Key Laboratory of Spring Rapeseed Genetic Improvement of Qinghai Province, Laboratory for Research and Utilization of Qinghai Tibet Plateau Germplasm Resources, National Key Laboratory Breeding Base for Innovation and Utilization of Plateau Crop Germplasm, Xining 810016, China; Academy of Agricultural and Forestry Sciences, Qinghai University, Xining 810016, China; Key Laboratory of Spring Rapeseed Genetic Improvement of Qinghai Province, Laboratory for Research and Utilization of Qinghai Tibet Plateau Germplasm Resources, National Key Laboratory Breeding Base for Innovation and Utilization of Plateau Crop Germplasm, Xining 810016, China; Academy of Agricultural and Forestry Sciences, Qinghai University, Xining 810016, China; Key Laboratory of Spring Rapeseed Genetic Improvement of Qinghai Province, Laboratory for Research and Utilization of Qinghai Tibet Plateau Germplasm Resources, National Key Laboratory Breeding Base for Innovation and Utilization of Plateau Crop Germplasm, Xining 810016, China; Academy of Agricultural and Forestry Sciences, Qinghai University, Xining 810016, China; Key Laboratory of Spring Rapeseed Genetic Improvement of Qinghai Province, Laboratory for Research and Utilization of Qinghai Tibet Plateau Germplasm Resources, National Key Laboratory Breeding Base for Innovation and Utilization of Plateau Crop Germplasm, Xining 810016, China; Academy of Agricultural and Forestry Sciences, Qinghai University, Xining 810016, China; Key Laboratory of Spring Rapeseed Genetic Improvement of Qinghai Province, Laboratory for Research and Utilization of Qinghai Tibet Plateau Germplasm Resources, National Key Laboratory Breeding Base for Innovation and Utilization of Plateau Crop Germplasm, Xining 810016, China

## Abstract

Spring-type *Brassica rapa* L. is a valuable genetic resource for breeding early-maturing crops, offering advantages such as early flowering and rapid maturation. However, the genetic mechanisms governing flowering time in spring-type *B. rapa* remain insufficiently understood. In this study, we investigated the flowering-time trait of an extremely early-maturing landrace, ‘Haoyou 11’, originating from the Qinghai-Tibetan Plateau. Initial mapping was conducted using an F_2_ population derived from the cross between Haoyou 11 and Dahuang (a late-flowering spring-type landrace of *B. rapa*). A major quantitative trait locus for flowering time, designated *qFTA06*, was identified within a 1.7-Mb interval on chromosome A06 using genotyping-by-sequencing and bulked segregant analysis sequencing (BSA-seq). The locus *qFTA06* was subsequently fine-mapped to a 75.16-kb region with a set of near-isogenic lines (NILs), and *BrCDF3*, a gene encoding a Dof transcription factor, was identified as the causal gene underlying *qFTA06*. Virus-induced gene silencing experiments revealed that *BrCDF3* acts as a negative regulator of flowering time under long-day conditions, with sequence variation contributing to the early-flowering phenotype in Haoyou 11. Phenotypic analysis of NILs showed that NIL-E, carrying the *BrCDF3* allele from Haoyou 11, flowered ~7 days earlier than NIL-L, which harbors the *BrCDF3* allele from Dahuang. By employing CRISPR/Cas9 technology, we further validated that the homologous gene *BnCDF3* also functions as a negative regulator of flowering time in *Brassica napus* L., and analyzed natural variations in the *CDF3* gene across natural populations. This study provides new insights into the genetic basis of flowering time in spring-type *B. rapa*, advancing early-maturity breeding efforts in crops.

## Introduction

Early maturity is a critical objective in crop breeding. The cultivation of early-maturing crops is an effective strategy to increase the total annual yield in countries with multiple cropping systems [1]. In southern China, the multiple cropping system of vegetables (e.g. early-maturing *Brassica rapa*) and cereals (e.g. rice and maize) has improved the multiple cropping index of land while optimizing the utilization of natural resources [2–4]. In high-altitude and high-latitude regions, the growth of early-maturing varieties helps mitigate low-temperature stress and frost damage. Spring-type *B. rapa* and *Brassica napus* are primarily cultivated in high-altitude regions of western China and high-latitude areas in the north as a summer crop, where cooler temperatures and shorter frost-free periods demand early-maturing varieties [5]. Previous studies have demonstrated a highly significant correlation between initial flowering times and the maturity period in early-maturing strains, as assessed through correlation evaluations [6, 7]. Breeders often use initial flowering time as a selection criterion to tailor flowering traits to local climate conditions [8, 9].

Within the *Brassica* genus, *B. rapa* ssp. *oleifera* accessions (AA, 2*n* = 20) flower earlier than any other species in U’s *Brassica* triangle, making them a valuable genetic resource for breeding early-maturing varieties [10, 11]. Additionally, the high compatibility between *B. napus* and *B. rapa* provides favorable conditions for interspecific hybridization [12]. This hybridization has been employed to transfer early-maturity traits from *B. rapa* into *B. napus*, addressing the limited availability of early-maturing resources and enhancing genetic diversity [5, 13, 14]. Notably, the Qinghai-Tibet Plateau, one of the original regions of *B. rapa* ssp. *oleifera*, harbors abundant germplasm resources of *B. rapa* [15]*.* Haoyou 11, a *B. rapa* landrace derived from Menyuan County, Qinghai Province, is one of the world’s earliest maturing resources. When spring-sown in Xining, Qinghai Province, Haoyou 11 flowers within ~30 days and has a growth period of about 90 days. Recognized for its extremely early maturity and stress resistance, Haoyou 11 is a valuable germplasm resource extensively utilized in breeding early-maturing *B. rapa* and *B. napus*. Compared to conventional early-maturing *B. rapa* and *B. napus* varieties, Haoyou 11 exhibits superior adaptability, allowing cultivation at altitudes above 2700 meters with short frost-free periods [16]. Conventional varieties, with longer growth cycles, fail to mature in these high-altitude areas, making Haoyou 11 irreplaceable in such environments [17]. Despite its significance, the molecular mechanism of the early-flowering trait of Haoyou 11 is still unknown. Identifying genes associated with its early flowering and maturity traits and detecting functional variants could expedite breeding progress and provide a theoretical foundation for cultivating early-maturing varieties.

Given the critical role of flowering time in breeding and genetic improvement, unraveling the molecular mechanisms underlying these traits has become a key research focus. The genome of *B. rapa* has experienced a triplication event, resulting in a more complex regulatory mechanism compared to that of *Arabidopsis thaliana* [18]. Two primary strategies are used to identify flowering-time genes in *B. rapa*: homologous cloning and positional cloning (including linkage and association mapping). Through homologous cloning, Schranz *et al*. isolated four *FLC* homologs: *BrFLC1*, *BrFLC2*, *BrFLC3*, and *BrFLC5* [19]; Jung *et al*. identified 223 flowering-related genes in *B. rapa* based on 174 flowering genes from *Arabidopsis*, including *BrFLC1/2/3/5*, *BrMAF*, *BrCOL1-2*, *BrFT1/2*, and *BrSOC1/2/3*, which showed strong responses to vernalization [20]; and Gao *et al*. performed genome-wide association analysis and preliminarily identified 14 candidate genes associated with flowering time in *B. rapa*, including *FT*, *TFL1*, and *CDF2* [21]. Although numerous flowering-time-related genes have been identified, research has primarily focused on the functional characterization of a few key genes, such as *FLC*, *FT*, *FRI*, and *SOC1* [22–26], in vegetable-type *B. rapa*, while studies on spring-type *B. rapa* remain poorly explored.

In flowering regulation, the expression of flowering-related genes is predominantly governed by transcription factors (TFs) [27]. Several TF families, including MADS-box, PEBP, CCT, and Dof, are known to regulate flowering [27, 28]. Among these, Dof (DNA-binding with one finger) TFs have emerged as pivotal components of the flowering regulatory network, modulating photoreceptor signal transduction, hormonal responses, and flowering induction [29–31]. Dof proteins, characterized by a conserved Dof domain at their N-terminus, exhibit dual functionality, acting as activators or repressors depending on their target genes [32]. These Dof factors can either suppress or activate transcription, depending on the specific target genes [29]. The Dof domain comprises 50 amino acids and enables both DNA binding and protein–protein interactions [32, 33]. Studies in *Arabidopsis* have revealed that the D subfamily of Dof genes plays a critical role in regulating flowering time [31, 34]. A subset of these genes, referred to as *CYCLING DOF FACTORS* (*CDFs*), exhibits circadian rhythm-dependent oscillations in expression [35, 36]. Under long-day (LD) conditions, overexpression of *CDF1* and *CDF3* driven by the cauliflower mosaic virus 35S promoter (CaMV35S), as well as *CDF2*, *CDF3*, *CDF4*, *COG1*, *CDF5*, and *CDF6* driven by the *SUCROSE TRANSPORTER 2* (*SUC2*) promoter, delayed flowering in *Arabidopsis* [35–38]. In contrast, no changes in flowering time were observed under short-day (SD) conditions. *Arabidopsis* quadruple mutant *cdf1-R cdf2-1 cdf3-1 cdf5-1* exhibited early flowering, independent of photoperiod [36]. These findings indicate that CDFs redundantly suppress the expression of flowering activation genes such as *CO* and *FT* within the photoperiod pathway [36]. Studies have also identified CDFs in other crops, including rice (*OsDof12*, *OsDof4*) [39, 40], tomato (*SlCDF1*–*5*) [41], jatropha (*JcDof1, JcDof3*) [42], potato (*StCDF1*) [43], and rapeseed (*BnCDF1*) [44], which are implicated in flowering regulation through photoperiod pathways. Current research on the CDF family predominantly focuses on *CDF1* and *CDF2* [35, 36], whereas the molecular mechanisms underlying *CDF3*-mediated flowering regulation remain relatively understudied. In *Arabidopsis*, *CDF3* expression is regulated by the photoperiod and exhibits circadian rhythm patterns, enabling it to modulate *CO/FT* gene expression either directly or indirectly [36]. Yeast two-hybrid and pull-down assays have confirmed that AtCDF3 interacts with FKF1 and LKP2 [35]. Furthermore, it has been reported that the transcriptional repressor PRR family members—PRR5, PRR7, and PRR9—bind to the promoter regions of *AtCDF* genes (e.g. *AtCDF2*, *AtCDF3*, and *AtCDF5*) in the afternoon, thereby regulating their transcriptional activity [45]. In *tomato*, the *SlCDF3* gene is also regulated by the circadian clock, and its functional mechanism is comparable to that of *AtCDF*. Notably, the overexpression of *SlCDF3*, but not *SlCDF1*, in *Arabidopsis* significantly reduces the expression levels of *CO* and *FT*, resulting in delayed flowering [46]. Recent studies have identified the *RsCDF3-RsVRN1* module in *radish*, which regulates bolting and flowering via vernalization pathways, rather than in a *CO/FT*-dependent manner. *RsCDF3* directly binds to the *RsVRN1* In-536 allele, inhibiting its transcriptional activity and suppressing flowering [47].

Despite advances in identifying flowering-related genes in *B. rapa* and *B. napus*, studies on extremely early-flowering landraces and the development of novel high-quality varieties remain limited. Investigations of the Dof TFs in these species are particularly sparse, with only *BnCDF1* having been isolated and functionally characterized to date [44]. In our study, we developed a segregation population with Haoyou 11 and Dahuang to dissect the genetic bases of flowering time in *B. rapa*. Map-based cloning identified *BrCDF3*, a Dof TF-encoding gene, to be the causal gene for *qFTA06*. Virus-induced gene silencing (VIGS) and CRISPR/Cas9 experiments demonstrated that *CDF3* negatively regulates flowering time in *B. rapa* and *B. napus*. Moreover, haplotype analysis revealed functional variations that influence flowering time phenotypes in natural populations. These findings highlight the pivotal role of *CDF3* in flowering regulation and underscore the potential of Dof TFs in breeding early-maturing *Brassica* varieties. This study provides a theoretical basis for accelerating early-maturing breeding.

**Figure 1:**
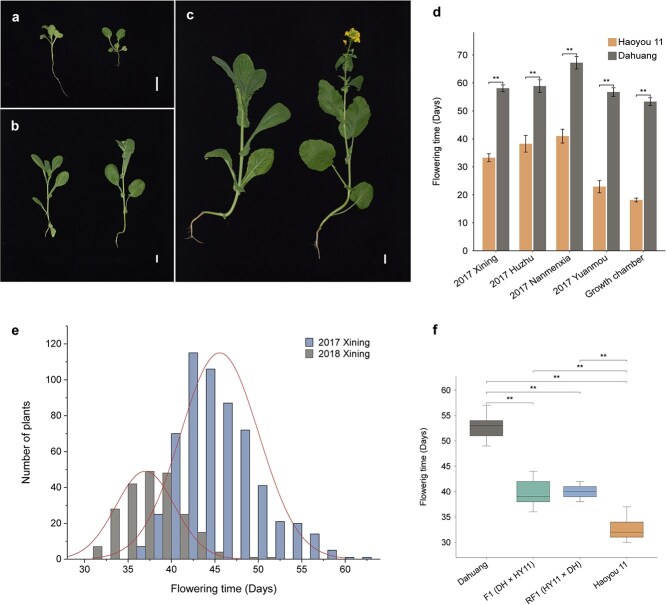
The flowering time phenotypes of Dahuang, Haoyou 11, F_1_, RF_1_, and F_2_ populations under different environments. (a–c) The phenotypes of Dahuang (left) and Haoyou 11 (right) at various growth stages in the plant growth chamber. Bars = 1 cm. (a) The seedling stage of Dahuang and Haoyou 11 (7 days post-sowing). (b) The seedling stage of Dahuang and the bud stage of Haoyou 11 (14 days post-sowing). (c) The bud stage of Dahuang and the flowering stage of Haoyou 11 (21 days post-sowing). (d) Comparative analysis of flowering time between parental lines across diverse environmental conditions. (e) Frequency distribution of flowering time in F_2_ populations under the spring environment in 2017 and 2018. The F_2_ population was planted in April 2017, consisting of 585 individual plants, and in May 2018, comprising 220 individual plants. (f) Comparison of flowering time among Dahuang, Haoyou 11, F_1_, and RF_1_ plants under the spring environment in 2018. All data in (d) and (f) are shown as mean ± SD. Error bars represent standard deviations. ^**^P < 0.01 (*t*-test).

## Results

### Phenotypic characterization and inheritance of the flowering time trait

The flowering time of Dahuang and Haoyou 11 was analyzed in an artificial climate chamber and across four field locations (Xining City, Huzhu County, Nanmenxia Weiyuan Town, and Yuanmou County) in 2017 (Fig. 1a–d, Table S1). Significant differences in flowering time were observed between the two parents in all environments, with Haoyou 11 flowering 20.63–35.19 days earlier than Dahuang. Notably, Haoyou 11 flowered as early as 18.14 days after seedling emergence in a controlled growth chamber. In Yuanmou, Haoyou 11 flowered 22.92 days post-emergence, whereas in Xining under spring conditions, it flowered after 33.27 days. In higher-altitude regions such as Huzhu and Nanmenxia, flowering occurred ~38.24–41.00 days after emergence. These results highlight the critical role of temperature and light in influencing the flowering time of Haoyou 11. In contrast, Dahuang, the late-flowering parent, required more than 50 days to flower in all environments. Morphological differences between Dahuang and Haoyou 11 were not apparent from the cotyledon stage to the two-leaf stage. However, distinct phenotypic differences emerged at the bolting stage, which were visible to the naked eye (Fig. 1a–c).

In 2018, the flowering time of the parents, F_1_, and RF_1_ hybrids were investigated. The flowering times of F_1_ and RF_1_ were intermediate between those of the two parents, showing a tendency toward the early-flowering parent, Haoyou 11 (Fig. 1f). Specifically, F_1_ flowered 6.93 days later than Haoyou 11 and 12.92 days earlier than Dahuang, while RF_1_ flowered 7.18 days later than Haoyou 11 and 12.67 days earlier than Dahuang (Table S2). Statistical analysis revealed no significant difference in flowering time between F_1_ and RF_1_, indicating that the flowering time trait is primarily controlled by nuclear genes (Fig. 1f). The flowering time of the F_2_ population exhibited a continuous and approximately normal distribution across two environments (2017 Xining and 2018 Xining), suggesting that the flowering time trait of Haoyou 11 is a quantitative trait regulated by multiple loci. This makes Haoyou 11 an ideal model for quantitative trait locus (QTL) analysis (Fig. 1e). Variance analysis (ANOVA) was performed on the flowering time traits of two parents, F_1_ hybrids, and F_2_ populations. The broad-sense heritability (*h*${}_B^2$) of flowering time was calculated to be 81.18%, further supporting the genetic basis of this trait.

### Construction of a high-density linkage map

Genotyping-by-sequencing (GBS) was conducted on an F_2_ population consisting of 203 progenies and the two parent lines, Haoyou 11 and Dahuang, to construct a high-density genetic linkage map. After sequencing and filtering, a total of 19.41 million clean paired-end (PE) reads (Q20 ≥ 98.52%, Q30 ≥ 95.24%) with a length of 150 bp were obtained for Haoyou 11, and 17.78 million clean PE reads (Q20 ≥ 97.67%, Q30 ≥ 92.24%) were obtained for Dahuang (Table S3). The guanine-cytosine (GC) content was 37.83% in Haoyou 11 and 42.45% in Dahuang. For the 203 F_2_ individuals, 97.59 Gb of clean sequences were generated, ranging from 247.59 Mb to 1006.64 Mb, with an average of 533.55 Mb clean data per individual (Q20 ≥ 96.26%, Q30 ≥ 93.92%). The mapping rate of clean read varied between 81.76% and 93.81%, with an average depth of 8× across the offspring (Table S4). A total of 154 539 homozygous mutation sites were identified between Haoyou 11 and Dahuang, distributed across 10 chromosomes. These included 129 944 SNPs and 24 595 InDels (Table S5). A 100-kb sliding window was used to analyze the genomic distribution of these variants (Fig.  S1). Among the SNPs, 75 608 were classified as transitions (A/G and C/T), and 54 336 as transversions (A/C, A/T, C/G, and G/T), resulting in a transition-to-transversion ratio (Ts/Tv) of 1.39 (Fig.  S2). The distributions of SNP types and InDel lengths are detailed in Figs  S2 and S3. Of the SNPs, 30.23% were located in intergenic regions, 18.31% in introns, 25.38% in exons, and 19.91% in upstream/downstream regions; for InDels, 25.95% were located in intergenic regions, 29.14% in introns, 6.42% in exons, and 26.76% in upstream/downstream regions (Tables S6 and S7). Within coding regions, we identified 19 980 synonymous SNPs, 12 748 nonsynonymous SNPs, 618 nonframeshift InDels, and 914 frameshift InDels (Tables S6 and S7). These SNPs and InDels were utilized for constructing the linkage map.

**Figure 2:**
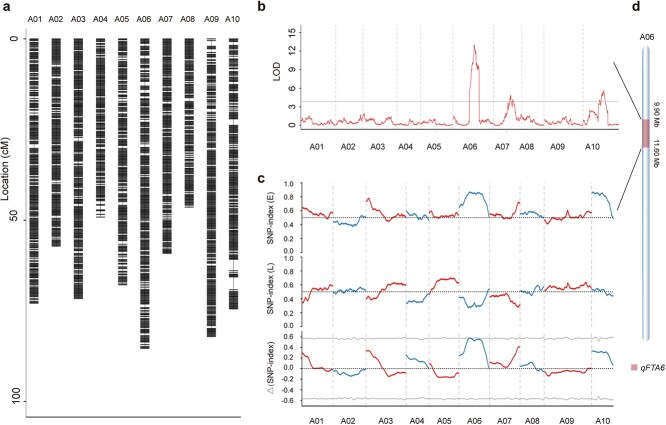
QTL mapping of the flowering time trait by genetic linkage mapping and BSA in the F_2_ population. (a) High-density genetic map of *Brassica rapa* L. The x-axis represents chromosome numbers, and the y-axis denotes genetic distances (cM); the black short lines indicate markers. (b) QTL detection of flowering time based on the GBS genetic map. The vertical axis represents the logarithm of odds (LOD) values, and the horizontal axis corresponds to distinct LG numbers. The gray horizontal line illustrates the threshold established through permutation testing. (c) QTL analysis of flowering time based on BSA-seq. The plot illustrates the distribution of Δ(SNP-index) across the 10 chromosomes. The red and blue curves represent the average value of SNP-index/Δ(SNP-index) within each window; the X-axis represents chromosome length (Mb); the three Y-axes (from top to bottom) represent the SNP-index (E), the SNP-index (L), and the Δ(SNP-index). The gray horizontal line indicates the threshold at the 99% confidence interval. (d) Location of the *qFTA06* locus on the Chiifu v3.5 genome according to QTL mapping based on GBS and BSA.

After removing unlinked markers, 151 718 markers were successfully mapped onto 10 linkage groups (LGs), corresponding to their respective chromosomes (Fig. 2a, Table S8). The completed linkage map encompassed a total length of 666.19 cM, featuring an average marker distance of 0.005 cM. The smallest LG, A08, contained 12 503 markers spanning a length of 46.40 cM, while the largest LG, chromosome A06, included 14 913 SNPs over a length of 85.35 cM. The maximum gaps in the LGs ranged from 0.75 cM (A02 and A07) to 3.57 cM (A10). Collinearity analysis demonstrated that the markers aligned with the genome exhibited high consistency, indicating robust collinearity and precise estimation of genetic recombination rates (Fig. S7).

### QTL analysis of flowering time in the F_2_ population

The QTL analysis of the flowering time trait was conducted in 203 individual plants from the F_2_ population using simplified genomic sequencing data. Permutation tests (PTs) were used to calculate the LOD thresholds for each LG. Composite interval mapping (CIM) was then performed, with results filtered using an LOD > 3.83. Three QTLs associated with flowering time were identified on chromosomes A06, A07, and A10, designated as *qFTA06*, *qFTA07*, and *qFTA10*, respectively (Fig. 2b, Table S9). The *qFTA06* was located between the flanking SNP markers c06b141 and c06b152 at positions 44.20 to 46.95 cM, with an LOD score of 13.00. This QTL exhibited an additive effect of 2.92 and explained 22.80% of the phenotypic variance. The QTL *qFTA07*, flanked by markers c07b125 and c07b135, was identified at positions 33.96 to 36.46 cM with an LOD score of 4.92. It explained 8.00% of the phenotypic variance and showed an additive effect of 0.763. The QTL *qFTA10*, flanked by markers c10b121 and c10b144, was detected at positions 38.84 to 45.32 cM, with an LOD score of 5.59. This QTL accounted for 10.60% of the phenotypic variance and demonstrated an additive effect of 1.8346. The genetic interval of *qFTA06* corresponded to a physical interval of 9.90 to 19.76 Mb on the Chiifu v3.5 genome, with Dahuang providing the allele associated with delayed flowering time.

To enhance reliability, the flowering time trait was further analyzed using phenotypic data from two extreme pools (23 early-flowering and 23 late-flowering individuals) within the F_2_ population (585 plants) combined with QTL-seq. A total of 38.01 Gb of resequencing data (Q20 ≥ 93.96%, Q30 ≥ 86.88%) was obtained from the two parents and the extreme phenotype pools, with GC content ranging from 38.04% to 42.45% (Table S10). The reference genome size was 391.41 Mb, and alignment rates ranged from 93.87% to 95.59%. The average read depths were 10.96× for Haoyou 11, 11.72× for Dahuang, 24.55× for the early-flowering pool, and 25.18× for the late-flowering pool. Variant detection was performed using the GATK software. Following stringent filtering, a total of 1 305 571 homozygous mutation sites were identified between the two parental lines. The genomic distribution of all variations is illustrated in Fig.  S4. These variations comprised 1 064 465 SNPs and 241 106 InDels (Table S11). The classification of SNP types and the length distribution of InDels are detailed in Figs  S5 and Figure  S6. Among the SNPs, 601 483 were transitions (A/G and C/T), and 462 982 were transversions (A/C, A/T, C/G, and G/T), yielding a transition/transversion ratio (Ts/Tv) of 1.30 (Fig.  S5). Annotation of SNPs and InDels was conducted using the ANNOVAR software. The results indicated that 20.06% of SNP sites were located in intergenic regions, 22.72% in introns, 33.02% in exons, and 17.31% in upstream/downstream regions; for InDels, 14.54% were in intergenic regions, 40.03% in introns, 8.41% in exons, and 23.05% in upstream/downstream gene regions (Tables S12 and S13). In coding regions, we identified 219 463 synonymous SNPs, 120 601 nonsynonymous SNPs, 8179 nonframeshift InDels, and 11 562 frameshift InDels (Tables S12 and S13). The Δ(SNP-index), computed as the difference between the SNP index values of the two pools, was plotted relative to the Chiifu genome. One candidate interval was identified where the mean line surpassed the threshold (1000 PTs, 99% confidence interval): A06, 9.12 to 11.60 Mb (Fig. 2c, Fig.  S8, Table S14).

BSA-seq combined with the genetic linkage map pinpointed the 9.90 to 11.60 Mb region on chromosome A06 as the primary effective QTL controlling flowering time, designated as *qFTA06* (Fig. 2d).

### Fine-mapping of *qFTA06* to a 75.16-kb region

**Figure 3:**
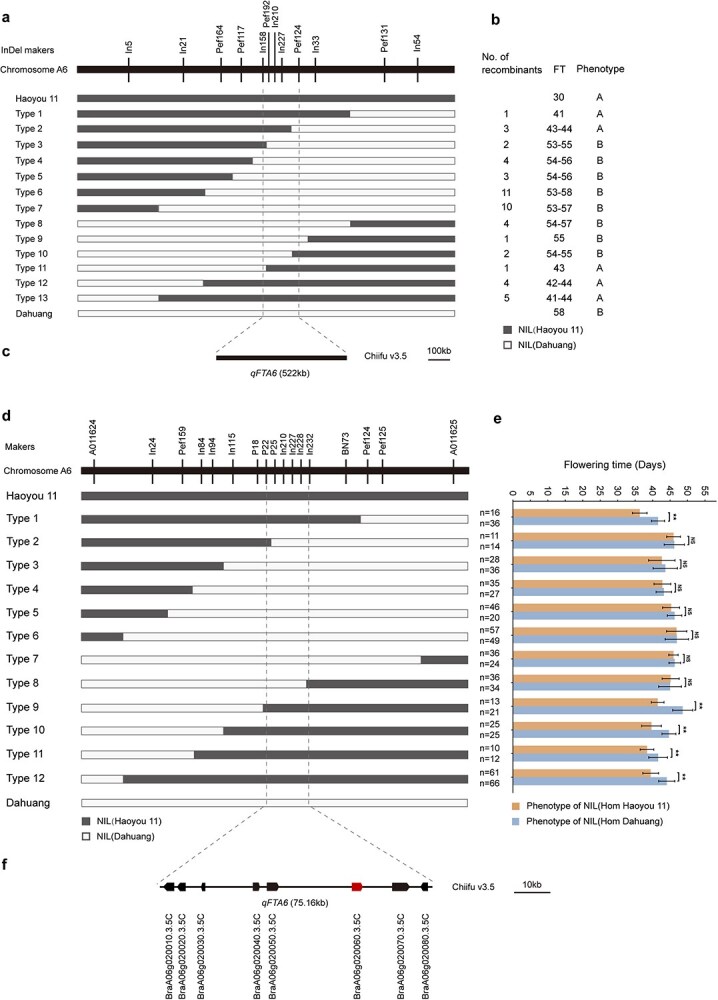
Fine mapping of *qFTA06* locus. (a) Genotypes of the two parents and recombinants with extreme flowering times selected from a BC_3_F_3_ population. Black short lines represent molecular markers within the candidate interval. Gray bars/NIL (Haoyou 11) indicate the genotypes of Haoyou 11, and white bars/NIL (Dahuang) indicate the genotypes of Dahuang. (b) Phenotypes of the two parents and recombinants with extreme flowering times selected from a BC_3_F_3_ population. The number of recombinants refers to the number of extreme recombinant individuals, and FT indicates flowering time. A represents the extreme early-flowering phenotype, and B denotes the extreme late-flowering phenotype. (c) The 522-kb *qFTA06* interval on the Chiifu v3.5 genome delimited by markers In158 and Pef124. (d) Genotypes of the two parents and recombinant plants from two BC_3_F_3_ populations. (e) Progeny testing of twelve representative recombinants. ‘n’ represents the number of recombinant progeny used for progeny testing. Orange bars indicate the phenotype of Haoyou 11 homozygous segments, and blue bars indicate the phenotype of Dahuang homozygous segments. Data are presented as mean ± SD. Error bars indicate standard deviations. ^**^P < 0.01 (*t*-test). NS denotes no significance. (f) Annotated genes within the 75.16-kb region in *B. rapa* (Chiifu v3.5). Annotated genes are represented by rectangles with arrows. Arrows signify the direction and position of transcription.

To further refine the *qFTA06* locus, secondary segregating populations were developed (Fig.  S9), and seven flanking markers (A011624, A011625, BN900073, Pef83, Pef85, Pef87, Pef93) were employed for foreground selection (Fig.  S10, Table S15). The BC_3_F_3_ populations were grown in a greenhouse and experimental field in 2019 and 2020, respectively, to identify recombinants. In 2019, a BC_3_F_3_ population comprising 840 individual plants from five lines was planted in the greenhouse. Using kompetitive allele-specific PCR (KASP) markers A011624 and A011625 flanking the target interval, 69 recombinants were identified, and their self-pollinated progeny were tested in 2021. In 2020, a BC_3_F_3_ population of 2236 individuals derived from five lines was sown in the experimental field. Genotyping with flanking markers identified 208 recombinant lines, which were self-pollinated to generate BC_3_F_4_ populations. The flowering time phenotypes of these lines were assessed, and 50 extreme phenotype recombinants were selected for further analysis. Subsequently, 12 InDel markers (In5, In21, Pef164, Pef117, In158, Pef192, In210, In227, Pef124, In33, Pef131, and In54) within the target interval were used for genotyping (Fig. 2a, Table S15). Clear electrophoretic bands were selected to genotype the recombinant plants with extreme phenotypes. Based on genotypes and recombination event locations, the 50 recombinant lines were classified into 13 types. Among them, markers Pef192, In210, and In227 co-segregated with the target gene (Fig. 3a and b). The region was narrowed down to a segment between markers In158 and Pef124, corresponding to 522 kb on chromosome A06 in the *B. rapa* genome (Chiifu v3.5) (Fig. 3c).

Using newly developed InDel markers associated with *qFTA06*, 277 recombinants were genotyped. Seventeen representative InDel markers were selected, and all recombinants were classified into 12 types based on their allele compositions and recombination breakpoints (Fig. 3d). In 2021, the recombinant progeny BC_3_F_4_ population was planted in the experimental field, and the phenotypes and genotypes of the offspring were recorded. Progeny testing validated the preliminary fine-mapping results, narrowing the *qFTA06* locus further to the region between InDel markers P22 and In232 (Fig. 3d–f). Markers P25, In210, In227, and In228 co-segregated with the target gene. Sequence alignment analysis revealed that this candidate interval corresponds to a physical region of ~75.16 kb on chromosome A06 of the *B. rapa* Chiifu v3.5 genome, which harbors five candidate genes (Fig. 3f).

### *BrA06CDF3* is the most likely candidate gene underlying *qFTA06*

**Figure 4:**
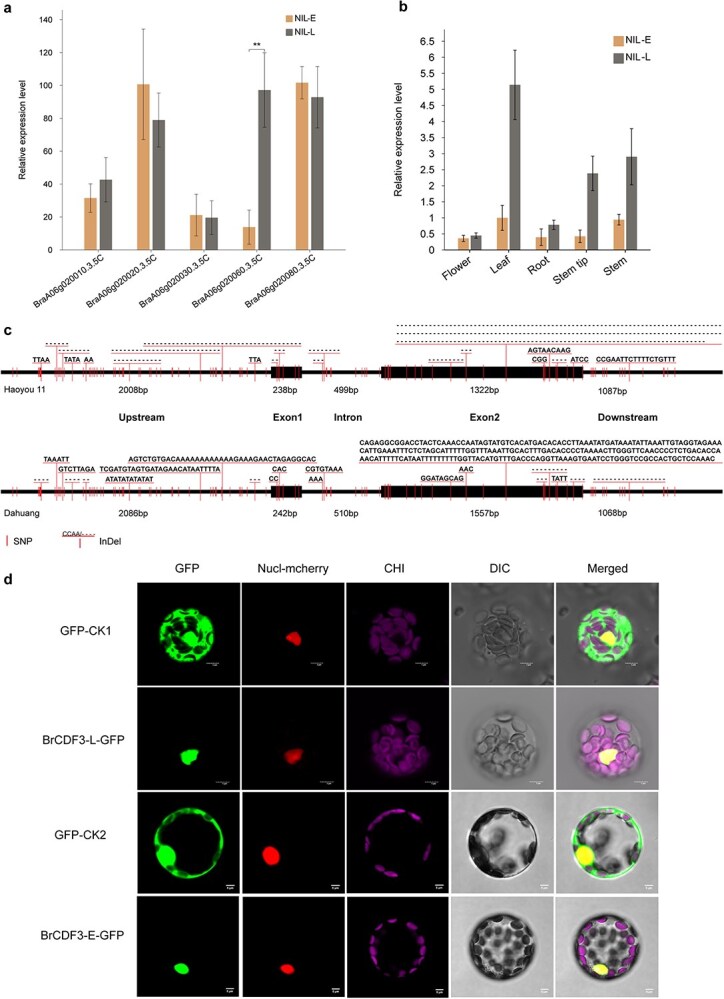
Sequence comparison and preliminary validation of *BraA06g020060.3.5C*. (a) Relative expression level of *BraA06g020060.3.5C* during the developmental stage. Orange bars on the left indicate the relative expression levels of NIL-E, and gray bars on the right denote the relative expression levels of NIL-L. Error bars show standard deviations. **P < 0.01 (Student’s *t*-test). (b) Expression pattern of *BrCDF3* in various tissues from NIL-E and NIL-L detected by qRT-PCR. (c) Gene structure and sequence comparison of *BraA06g020060.3.5C*. (d) Subcellular localization of free GFP (GFP-CK1 and GFP-CK2) and BrCDF3-L-GFP, BrCDF3-E-GFP fusion proteins in *Arabidopsis* protoplasts. Scale bars, 5 μm.

The *qFTA06* interval, spanning In22–In232, comprises eight candidate genes, three of which lack Arabidopsis homologs and functional annotations. Cross-referencing genome annotations from *B. rapa* (Chiifu v3.5), *B. napus* (ZS11 v0), and *Arabidopsis* (v10) validated five predicted protein-coding genes within this region (Fig. 3f, Table S16). Resequencing identified sequence variations in eight candidate genes (Tables S17 and S18). Variants were detected in seven genes, except *BraA06g020070.3.5C*. Due to substantial genomic divergence between Haoyou 11 and Dahuang, the causal gene could not be determined solely by candidate gene sequence analysis. The identified genes—*BraA06g020010.3.5C*, *BraA06g020020.3.5C*, *BraA06g020030.3.5C*, *BraA06g020060.3.5C*, and *BraA06g020080.3.5C*—encode homologs of the *Arabidopsis* zinc finger superfamily protein, MDH, transmembrane protein, CDF3, and HNH endonuclease, respectively (Table S16). Functional annotation indicated that only *CDF3* (*BraA06g020060.3.5C*), encoding a Dof TF, was associated with plant development and flowering time. The mRNA transcripts of all candidate genes were detected at the pre-flowering developmental stage, but only *BraA06g020060.3.5C* exhibited significantly different transcript levels between early-flowering NIL-E and late-flowering NIL-L (Fig. 4a). *BraA06g020060.3.5C* showed elevated transcript levels in NIL-L compared to NIL-E. These results strongly suggest that the *CDF3* gene is the most likely causal gene underlying *qFTA06* for the flowering time trait, subsequently designated as *BrCDF3*. We assessed the expression profile of *BrCDF3* using quantitative real-time PCR (qRT-PCR). *BrCDF3* exhibited ubiquitous expression in leaves, stem tips, stems, roots, and flowers, with preferential expression observed in leaves, stem tips, and stems in NILs (Fig. 4b).

Cloning and sequencing analysis revealed that *BrA06CDF3* consists of two exons and one intron in all parental lines and NILs (Fig. 4c). Full-length sequences of 5154 bp were amplified in the early-flowering lines Haoyou 11 and NIL-E, differing by only one SNP (Figs  S11 and Figure  S12). The sequence includes a 2008-bp promoter region, a 2059-bp coding region (exon 1: 238 bp; intron: 499 bp; exon 2: 1322 bp), and a 1087-bp downstream region (Fig. 4c). In the late-flowering lines Dahuang and NIL-L, the full-length gene was slightly longer at 5463 bp, differing by one SNP, and comprised a 2086-bp promoter region, a 242-bp exon 1, a 510-bp intron, a 1557-bp exon 2, and a 1068-bp downstream region (Fig. 4c, Figs  S11 and Figure  S13). Sequence alignment between early- and late-flowering lines identified 76 SNPs, 34 InDels, and a 236-bp structural variation (Fig. 4c, Figs S12–Figure S14). Among these, 13 mutations were non-synonymous and located in the coding region (Fig. S15). The upstream regulatory region (~2 kb) exhibited the greatest variation, with 39 SNPs and 18 InDels identified (Fig. S12).

Sequence analysis of the *BrCDF3* gene predicted that the encoded proteins, BrCDF3-E and BrCDF3-L, consist of 426 and 336 amino acid residues, respectively (Fig. S16). Both proteins possess a conserved Dof domain (IPR003851), spanning amino acids 107 to 163, and a region enriched in basic amino acids resembling bipartite nuclear localization signals, suggesting potential nuclear localization (Figs S16–Figure S18). To confirm the subcellular localization of BrCDF3, a 35S::BrCDF3-GFP construct was generated and transiently co-expressed with an mCherry-fused nuclear marker in *Arabidopsis* protoplasts. The overlap between eGFP and mCherry fluorescence confirmed that BrCDF3 localizes to the nucleus, consistent with database predictions (Fig. 4e). These findings support the role of BrCDF3 as a TF involved in flowering time regulation. To further investigate the phylogenetic relationship of BrCDF3 with homologous genes in other species, a BLASTP search was performed against the NCBI protein database using BrCDF3 as the query. The sequences of 16 homologous proteins were retrieved and used to construct a phylogenetic tree (Fig. S17). Phylogenetic analysis revealed that CDF3 is highly conserved within the *Brassicaceae* family, with BrCDF3 showing over 90% sequence similarity to BnA06CDF3.

### *CDF3* gene functions as a negative regulator for the flowering time of *B. rapa* and *B. napus*

**Figure 5:**
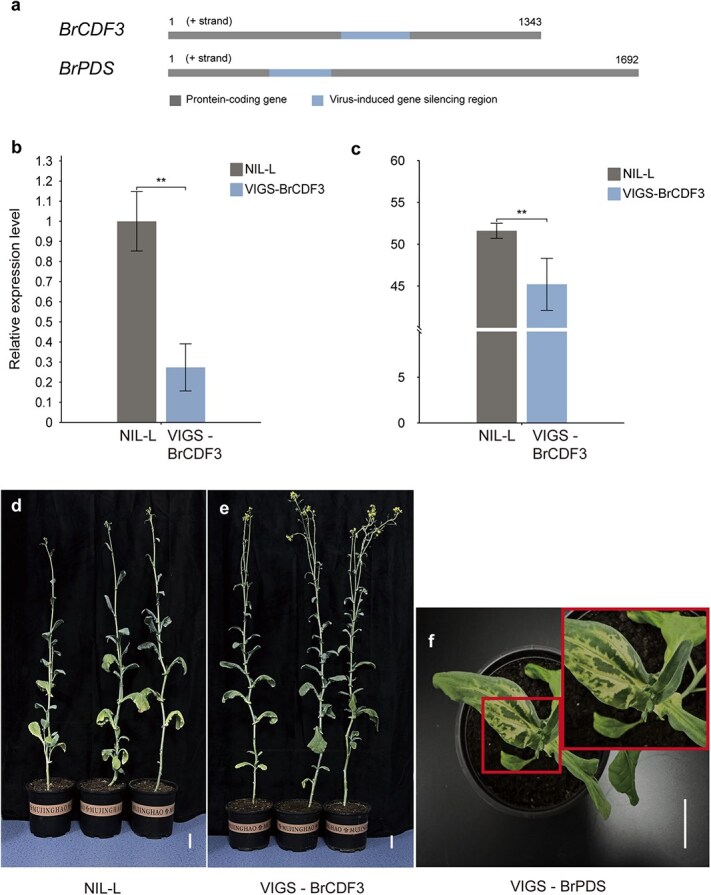
Functional verification of *BrCDF3* using virus-induced gene silencing. (a) Schematic representation of the specific target sequences for *BrCDF3* and *BrPDS*. (b) Relative expression levels of *BrCDF3* in the *BrCDF3*-silenced line and NIL-L. (c) Flowering time comparison between the *BrCDF3*-silenced line and NIL-L. Error bars represent standard deviations. ^**^P < 0.01 (Student’s *t*-test). (d–f) Phenotypic comparison of NIL-L, the *BrCDF3*-silenced line, and the *BrPDS*-silenced line. The *PDS*-silencing line was utilized as positive control. Scale bars = 5 cm.

To verify whether *BrCDF3* is involved in the regulation of flowering time in *B. rapa*, its expression was suppressed in NIL-L plants using VIGS. The target sequences of *BrCDF3* and *BrPDS* (Fig. 5a) were cloned into the pTRV2 vector to generate recombinant plasmids pTRV2::BrCDF3 and pTRV2::BrPDS. Each treatment included 50 individual plants: seedlings infiltrated with pTRV2::BrCDF3 served as loss-of-function lines, while non-infiltrated seedlings and those treated with the empty pTRV2 vector acted as negative controls. The phytoene desaturase (*PDS*) gene was used as a reporter to assess the effectiveness of the VIGS system. Due to the incomplete infection efficiency of VIGS, only plants exhibiting altered phenotypes and reduced gene expression were selected for statistical analysis. Five independent silenced lines displaying consistent phenotypic and growth characteristics were analyzed to assess the expression levels of *CDF3* and *PDS* at 21 days post-infiltration (dpi). Quantitative analysis revealed a significant reduction in *BrCDF3* transcript levels in the silenced lines compared to the control, with decreases ranging from 58.45% to 89.42% (Fig. 5b). Phenotypic observations demonstrated that *BrCDF3*-silenced plants flowered significantly earlier than control plants, with an average reduction of 6.40 days in flowering time (Fig. 5c–e). Despite variations in VIGS infiltration efficiency, all plants exhibiting markedly reduced *BrCDF3* expression showed early flowering, indicating that downregulation of *BrCDF3* in NIL-L accelerates flowering time. Additionally, at 21 dpi, *PDS*-silenced plants exhibited photobleaching in 8 out of 50 seedlings (Fig. 5f), confirming the effectiveness of the gene-silencing system employed in this study. Collectively, these findings provide strong evidence that *BrCDF3* is the causal gene regulating flowering time and functions as a negative regulator of floral transition in *B. rapa*.

To investigate whether the natural mutation in the *BrCDF3* gene of Haoyou 11 results in an early flowering phenotype and to further explore its role in regulating flowering time, the full-length sequence of *BrCDF3* from Haoyou 11 was introduced into wild-type *A. thaliana* (Col-0) via transgenic methods. Concurrently, *BrCDF3* alleles from both Haoyou 11 and Dahuang were overexpressed in the wild-type *Arabidopsis* background. Genetic complementation assay demonstrated that transgenic plants expressing the full-length *BrCDF3* sequence from Haoyou 11 exhibited a significantly earlier flowering time compared to the wild-type control. Four transgenic lines (*BrCDF3(E)*-Comp-1, *BrCDF3(E)*-Comp-2, *BrCDF3(E)*-Comp-6, and *BrCDF3(E)*-Comp-8) flowered significantly earlier than the wild-type control, suggesting that the natural mutation in *BrCDF3* from Haoyou 11 may be a key factor of the early flowering phenotype. Under the control of the 35S promoter, overexpression of *BrCDF3* from either Haoyou 11 or Dahuang resulted in delayed flowering. These findings collectively indicate that *BrCDF3* is a critical candidate gene at the *qFTA06* locus involved in flowering time regulation and functions as a negative regulator of flowering time. Moreover, the functional variation responsible for the early flowering phenotype in Haoyou 11 likely originates from non-coding regions of the gene, such as the promoter, 5’ UTR, 3’ UTR, or introns, rather than from variations within the coding sequence (CDS).

**Figure 6:**
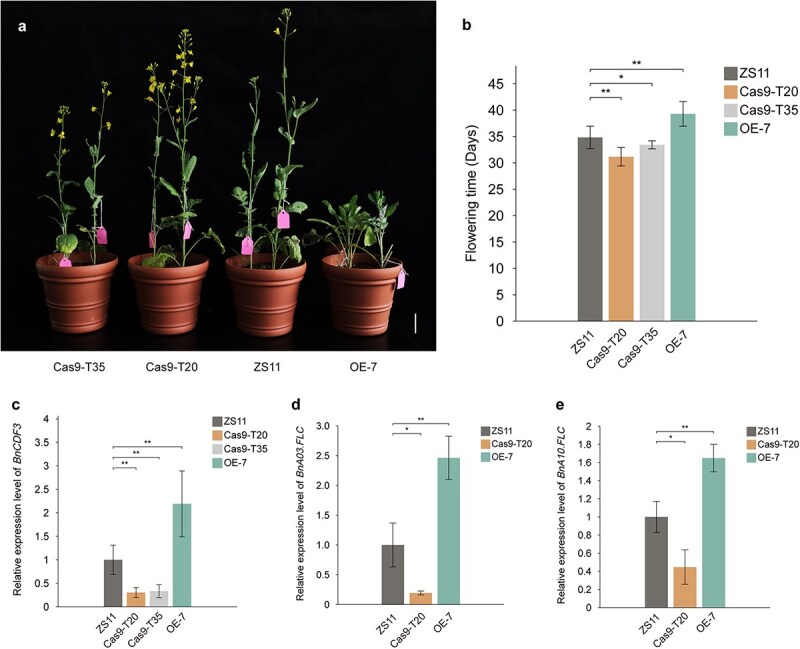
Functional validation of *BnCDF3* via overexpression and CRISPR/Cas9 gene editing. (a) Phenotypic comparison of transgene-positive T_1_ lines (Cas9-T20, Cas9-T35, OE-7) and wild-type ZS11. Scale bars = 5 cm. (b) Flowering times of transgenic T_1_ plants and ZS11. The vernalization period following seed germination was excluded from flowering time calculations. (c) Relative expression levels of *BnCDF3* in the transgenic lines and ZS11. (d) Relative expression levels of *BnA03.FLC* in the transgenic lines and ZS11. (e) Relative expression levels of *BnA10.FLC* in the transgenic lines and ZS11. Data in (b–e) were mean values ± SD. Error bars indicate standard deviations, and Student’s *t*-test was used to compare mutant lines and ZS11. ^*^P < 0.05, ^**^P < 0.01.

To further confirm the regulatory role of the *CDF3* gene in flowering time, we overexpressed *BnA06CDF3* and employed gene-editing technology to knockout both *BnA06CDF3* and its ortholog, *BnaC03CDF3*, in ZS11, a late-maturing rapeseed cultivar with normal *CDF3* expression levels. First, we developed transgenic T_1_ rapeseed plants constitutively expressing *BnA06CDF3* driven by the 35S promoter. Under LD conditions, the transgenic lines exhibited a significant delay in flowering compared to ZS11, with the OE-7 line flowering 4.45 days later than the WT (wild type) plants (Fig. 6a and b). Quantitative RT-PCR analysis further confirmed a significant increase in *BnA06CDF3* expression levels in the overexpression (OE) plants relative to ZS11 (Fig. 6c). Moreover, we selected plants with consistent growth and expression levels from both the OE-7 line and ZS11, and measured the relative expression of the flowering integrator genes *FT*, as well as the core flowering regulatory genes *CO* and *FLOWERING LOCUS C* (*FLC*). The results demonstrated that in transgenic plants, the expression levels of the two *FLC* copies, *BnA03.FLC* and *BnA10.FLC* (Fig. 6d and e), were significantly increased, whereas no significant differences were observed in the transcriptional levels of *CO* and *FT*.

**Figure 7:**
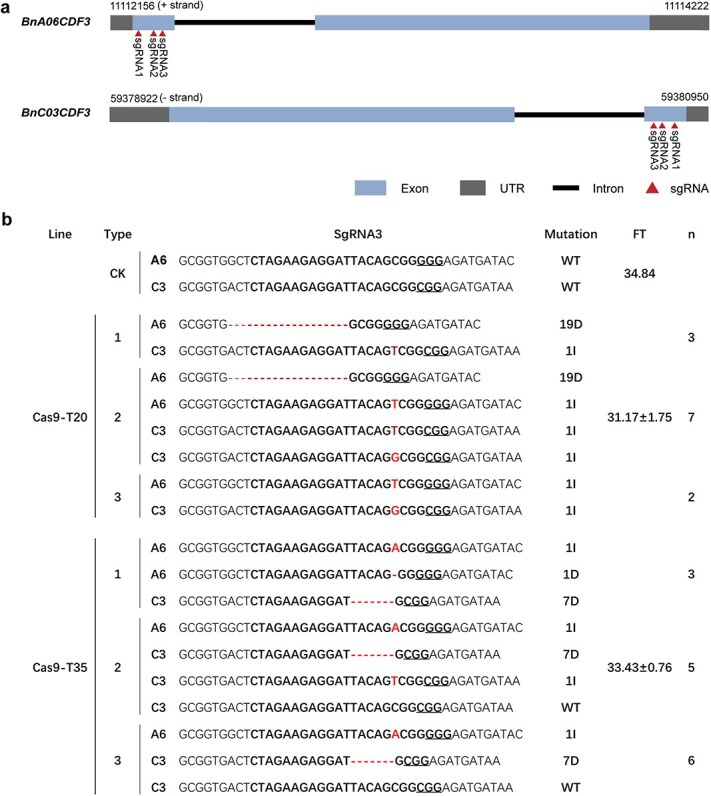
Sequence analysis and phenotypic evaluation of *BnCDF3* knock-out mutant lines in the T_1_ generation. (a) Gene structure of *BnCDF3* and the locations of three sgRNA. (b) Mutations and phenotypes of two *CDF3* mutant lines. The bold bases denote the target sequence. The red bases indicate changed bases. D, deletion; I, insertion.

To further investigate the functional role of *BnCDF3* in flowering time regulation, we employed CRISPR/Cas9-mediated gene editing. Three single-guide RNAs (sgRNAs) targeting the first exon of *BnCDF3* were designed (Fig. 7a). Sequence analysis of the edited plants revealed that all mutations were located at the sgRNA3 target site (Fig. 7b). Two independent transgenic T_1_ lines, Cas9-T20 and Cas9-T35, were selected, both carrying frameshift mutations in *BnA06CDF3* and *BnaC03CDF3*, leading to early flowering phenotypes. To validate the induced mutations, HT-F/R primers were used to obtain a 233-bp segment from chromosome A06 and a 240-bp region from chromosome C03, both containing the sgRNA target sites. High-throughput tracking of mutations (Hi-TOM) was subsequently employed to sequence the amplified products, allowing for precise identification of mutation patterns in *BnA06CDF3* and *BnaC03CDF3* (Fig. 7b). Flowering time phenotypes were analyzed in 12 transgenic plants from the Cas9-T20 line and 14 transgenic plants from the Cas9-T35 line (Fig. 6a). The results showed that Cas9-T20 and Cas9-T35 flowered 3.67 days and 1.40 days earlier, respectively, than ZS11, with significant differences observed (Fig. 6b). The qRT-PCR analysis showed that the expression level of *BnCDF3* was significantly reduced in gene-edited plants compared to ZS11 (Fig. 6c). Further investigation of the key downstream flowering genes also revealed no significant changes in the expression of *CO* and *FT*. The transcriptional levels of the two *FLC* homologs, *BnA03.FLC* and *BnA10.FLC*, were markedly decreased, leading to an advanced flowering time (Fig. 6d and e). These findings provide additional evidence that *BnCDF3* does not regulate bolting and flowering via the classical *CO/FT* transcriptional pathway, but instead by modulating the transcript levels of flowering gene *FLC*. These results strongly suggest that *BnCDF3* functions as a negative regulator of flowering time in *B. napus*, consistent with the role of *BrCDF3* in *B. rapa*.

### The influence of genetic variation within the *CDF3* gene on flowering time

**Figure 8:**
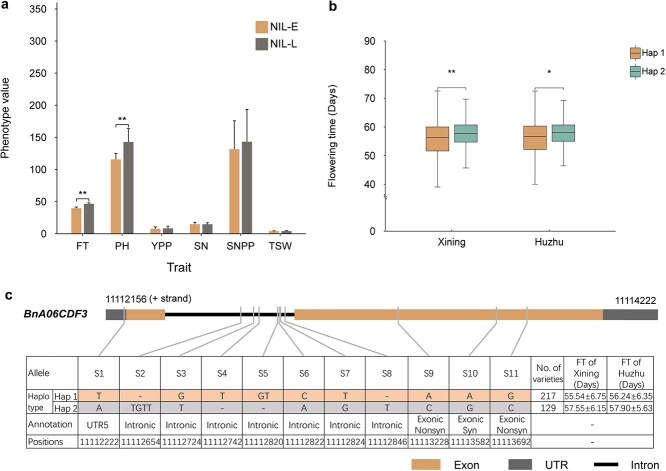
Evaluation of agronomic traits in NILs and haplotype analysis of *BnA06CDF3* in natural population. (a) Evaluation of agronomic traits in near-isogenic lines. FT: Flowering time, PH: Plant height, YPP: Yield per Plant, SN: Seed number per Silique, SNPP: Silique number per Plant, TSW: Thousand Seed Weight. (b) Influence of *BnA06CDF3* haplotypes on flowering time in 346 accessions grown under two different environmental conditions. (c) Haplotype classification and allelic variations of *BnaA06CDF3* in 346 accessions of *B. napus*. All data in (a–c) are presented as mean values ± SD. Error bars represent standard deviations. ^*^P < 0.05, ^**^P < 0.01 (*t*-test).

To investigate the potential application value of the *BrCDF3-E* (early flowering) gene in Haoyou 11, we developed a set of NILs using Dahuang as the receptor parent and evaluated the key agronomic traits of these NILs (such as flowering time, plant height, yield per plant, seed number per silique, silique number per plant, and thousand seed weight) (Table S22). Phenotypic analysis revealed significant differences in flowering time and plant height between the NILs: compared with the late-flowering NIL-L, the early-flowering NIL-E flowered ~6.87 days earlier, while the plant height of NIL-L was ~27.15 cm greater than that of NIL-E (Fig. 8a). Additionally, no significant differences were observed in yield-related traits among the NILs, indicating that under the same genetic background, the introduction of the early-flowering *BrCDF3-E* gene from Haoyou 11 had no substantial impact on yield (Fig. 8a, Table S21).

To identify additional functional variants of *CDF3* in natural populations, sequence polymorphisms in *BnA06CDF3* across 346 accessions of *B. napus* were analyzed using whole-genome resequencing data and Candihap software (Table S21). A total of 11 significant sequence variations (S1–S11) were identified within *BnA06CDF3* (Fig. 8c). Of these, S1 was located in the 5′ untranslated region (UTR), S2–S8 were positioned within introns, and S9–S11 were present in exons, with S9 and S11 resulting in non-synonymous mutations. Based on these 11 polymorphic sites, the natural population was classified into two major haplotypes: Hap 1 (T-GTGTCT-AAG) and Hap 2 (ATGTTT--AGTCGC) (Fig. 8c). Among the 346 accessions, 217 accessions carrying the Hap 1 allele (T-GTGTCT-AAG) exhibited significantly earlier flowering compared to the 129 accessions harboring the Hap 2 allele (ATGTTT–AGTCGC). To assess the phenotypic impact of these haplotypes on flowering time, we analyzed flowering data from the natural population grown under two distinct environmental conditions. *T-*test revealed significant differences in flowering time between the two haplotype groups (Fig. 8b). These results indicate that natural variations in *BnA06CDF3* contribute to flowering time differences and suggest that the regulatory function of *CDF3* in flowering is conserved across diverse genetic backgrounds.

## Discussion

Haoyou 11 exhibits valuable agronomic traits, including early flowering and stress tolerance, making it a potential genetic resource for early-maturing breeding [5]. However, the molecular mechanisms underlying the early-flowering trait of Haoyou 11 have yet to be fully elucidated. In our study, we applied a map-based cloning strategy to identify genes associated with flowering time in Haoyou 11 and successfully isolated *BrCDF3*, a member of the Dof TF family. The function of *CDF3* was validated using VIGS experiments and CRISPR/Cas9-mediated gene editing. Furthermore, agronomic trait evaluation and haplotype analysis provided additional evidence supporting the potential application of *CDF3* in breeding programs aimed at improving early-maturing cultivars.

Traditional QTL mapping methods have been constrained by the limited number of genetic markers available. However, advances in high-throughput sequencing technologies have facilitated the generation of large-scale genetic variation datasets, significantly enhancing the resolution of QTL studies. In previous studies, BSA-seq has been widely employed for QTL mapping due to its cost-effectiveness [48]. Additionally, secondary segregating populations have been utilized to develop high-density genetic linkage maps, integrating phenotypic data for more precise QTL identification [49–51]. In this study, we employed GBS and BSA-seq to analyze an F₂ population, resulting in the identification of a major QTL, *qFTA06*. We further detected a substantial number of SNP and InDel variants within this region (Fig. 2). These variations enhanced both the efficiency and accuracy of QTL mapping, while also providing a set of molecular markers that can facilitate fine mapping and molecular breeding efforts. To dissect complex quantitative traits, constructing near-isogenic lines (NILs) through marker-assisted selection (MAS) can significantly shorten breeding cycles and minimize interference from other genes [52]. In this study, we developed KASP markers for MAS, which accelerated genotyping (Table S15). Using Haoyou 11 as the donor parent and Dahuang as the recurrent parent, we successfully constructed NILs and narrowed the *qFTA06* region to a 75.16-kb interval on chromosome A06 (Fig. 3).

Compared with *Arabidopsis*, the regulation of flowering time in *B. rapa* is more complex, primarily due to the whole-genome triplication event [18]. Wang *et al*. identified 549 potential flowering regulatory genes in the *B. rapa* genome through homologous comparison with 306 flowering-related genes from *Arabidopsis*, most of which participate in multiple flowering pathways [53]. Jung e*t al*. reported 223 additional flowering-related genes in *B. rapa*, based on 174 genes from *Arabidopsis*, including *BrFLC1/2/3/5*, *BrMAF,* B*rCOL1-2*, *BrFT1/2*, and *BrSOC1/2/3*, all of which showed significant responses to vernalization [20]. Previous studies have identified four homologous *FLC* genes in *B. rapa* (*BrFLC1/2/3/5*) [19]. These four *BrFLC* genes share functional similarities with *AtFLC* in *Arabidopsis*, acting as flowering repressors and playing key roles in regulating flowering in *B. rapa* [22]. Although numerous flowering-time-related genes have been identified, research has primarily focused on the functional characterization of a few key genes, such as *FLC*, *FT*, *FRI*, and *SOC1* [22–26, 54], in non-heading *B. rapa* (*subsp. chinensis*) and heading *B. rapa* (*subsp. pekinensis*). To date, studies on spring-type *B. rapa* remain poorly explored, and no members of the Dof gene family have undergone functional characterization in this species. In this study, we report for the first time the cloning and validation of the *BrCDF3* gene using spring-type *B. rapa* as the experimental material. Functional annotation and quantitative expression analysis indicated that the candidate gene *BrCDF3* is a homolog of *AtCDF3* in *A. thaliana*. Sequence alignment revealed multiple genetic variations between Haoyou 11 and Dahuang (Fig. 4c), suggesting that natural sequence variation in *BrCDF3* contributes to the early-flowering phenotype observed in Haoyou 11.

Members of the Dof TF family, known as cycling DOF factors (CDFs), are regulated by the circadian rhythm and function as inhibitors of *CO* and *FT* expression through the photoperiodic pathway under LD conditions [35, 36, 42]. As negative regulators of flowering time, *CDF* overexpression typically delays flowering [35, 37, 41, 44, 55, 56]. However, in *Oryza sativa*, *OsDof12* and *OsDof4* overexpression result in early flowering under LD conditions but delayed flowering under SD conditions [39, 40]. In this study, we established a VIGS system in *B. rapa* (Dahuang) and successfully inhibited the expression of *BrCDF3* in NIL-L lines (Fig. 5). Under LD conditions, reduced *BrCDF3* expression accelerated flowering in plants exhibiting successful gene silencing, confirming that *BrCDF3* functions as a negative regulator of flowering time in *B. rapa*. This result aligns with earlier research findings in *A. thaliana* and *Solanum lycopersicum*. Furthermore, we cloned the *CDF3* gene from *B. napus* and validated its function through Agrobacterium-mediated overexpression and CRISPR/Cas9 gene editing, demonstrating that *BnCDF3* also acts as a negative regulator of flowering time in *B. napus* (Fig. 6). The qRT-PCR analysis suggested that *BnCDF3* may not rely on the classical *CO/FT*-mediated flowering regulatory pathway but regulates the transcript levels of flowering gene *FLC*, leading to the change of flowering time (Fig. 6d and e). Previous research has indicated that the *CDFs* (*CDF1, CDF2, CDF3,* and *CDF5*) function redundantly to suppress floral transition in wild-type *Arabidopsis*, with double mutants exhibiting additive effects [23]. Notably, the quadruple mutant *cdf1-R cdf2-1 cdf3-1 cdf5-1* displays significantly accelerated flowering under both LD and SD conditions [36]. In our study, we observed that flowering time differences between gene-edited plants and wild-type plants were less pronounced compared to overexpression lines (Fig. 6a and b). This phenomenon may be attributed to functional redundancy among *CDF* genes, where other family members partially compensate for the loss of function.

CDF3, a TF, plays a crucial role in regulating plant responses to abiotic stresses, including salt stress, drought, and extreme temperatures [37, 46, 57]. Overexpression of *CDF3* has been shown to enhance tomato biomass, improve nitrogen accumulation efficiency, and delay flowering [46, 58]. Current studies on the *CDF* family primarily focus on *CDF1* and *CDF2*, whereas the molecular basis of *CDF3*-mediated flowering control remains less understood [35, 36]. *CDF3* expression follows circadian rhythms under photoperiodic regulation and downregulates *CO* and *FT* when overexpressed in *Arabidopsis* and *tomato* [36, 46]. Interaction assays revealed that AtCDF3 associates with FKF1 and LKP2 [35], and PRR5/7/9 repress transcription by binding to *AtCDF* promoters [45]. Studies have shown that in *Arabidopsis*, *CDF3* is significantly upregulated in response to drought, salt stress, extreme temperatures, and abscisic acid treatment [37]. Furthermore, researchers have demonstrated that *CDF3* plays multiple regulatory roles in modulating flowering time and enhancing abiotic stress tolerance through its interaction with the *CBF/DREB2A*–*CRT/DRE* and *ZAT10/12* modules [37]. The overexpression of *SlCDF3* tomato, but not *SlCDF1*, in *Arabidopsis* significantly reduces the expression levels of *CO* and *FT*, resulting in delayed flowering [46]. In *radish*, the *RsCDF3–RsVRN1* module regulates bolting and flowering through vernalization rather than the *CO/FT* pathway, with RsCDF3 binding directly to the *RsVRN1* In-536 allele to inhibit transcription [47]. Consistent with findings in radish, we observed alterations in the expression levels of the key flowering gene *FLC*. Notably, not all *FLC* members exhibited differential expression between early-flowering and late-flowering materials. Analysis of *FLC* gene transcription revealed that *BrFLC1* and *BrFLC3*, located on chromosomes A10 and A03, exhibited higher expression in Dahuang than in Haoyou 11 (unpublished). These findings align with our results in transgenic *B. napus*: in *CDF3* overexpression lines, *BnA10FLC* and *BnA03FLC* expression was significantly upregulated compared with the control, whereas expression was markedly reduced in *CDF3* knockout lines (Fig. 6d and e). This study establishes a theoretical foundation for the application of the flowering gene and provides new insights into the genetic basis of flowering time in spring-type *B. rapa*. Nevertheless, two primary limitations remain that require further investigation in future research. First, the analysis was based solely on a segregating population derived from two parental lines, leading to the identification of a limited number of flowering genes. Given that Haoyou 11 is an extremely early-flowering variety, its flowering genes and genetic variants require more comprehensive exploration. Second, since stable homozygous transgenic mutants of higher generations have not yet been obtained, a systematic investigation into the molecular regulatory mechanisms of *BrCDF3* and *BnCDF3* remains incomplete and requires further study.

Flowering time is a critical determinant of crop maturity, and modifying flowering-related genes enables breeders to develop early-maturing rapeseed varieties that are adaptable to diverse agro-climatic conditions [9]. The natural variations in *BrCDF3* identified in Haoyou 11 represent a valuable genetic resource for breeding early-flowering cultivars. To explore its breeding potential, we constructed a set of NILs using Dahuang as the genetic background (Fig.  S5). Specifically, NIL-E carries the *BrCDF3* allele from Haoyou 11, while NIL-L carries the *BrCDF3* allele from Dahuang. Phenotypic analysis revealed significant differences in flowering time and plant height among the NILs (Fig. 8a). NIL-E flowered 6.87 days earlier than NIL-L (Table S21). However, no significant differences were observed in yield-related traits among the NILs (Fig. 8a, Table S21), indicating that the early-flowering *BrCDF3-E* allele from Haoyou 11 had no significant effect on yield under the same genetic background. These results suggest that the *BrCDF3* allele in Haoyou 11 holds potential for the development and genetic improvement of early-flowering varieties. By identifying haplotypes associated with desirable flowering traits, breeders can develop early- or late-maturing varieties suited to various climatic regions [59]. We conducted haplotype analysis on 346 *B. napus* accessions, which were divided into two haplotypes: Hap 1 (T-GTGTCT-AAG) and Hap 2 (ATGTTT--AGTCGC) (Table S21, Fig. 8b and c). Across both Xining and Huzhu environments, plants carrying Hap 1 flowered earlier than those harboring Hap 2 (Fig. 8b). These findings suggest that *CDF3* plays a key role in regulating flowering time in *B. napus*, making it a promising target for rapeseed breeding programs aimed at optimizing flowering time for specific environmental conditions.

## Materials and methods

### Plant materials and population construction

Two *B. rapa* lines, Haoyou 11 and Dahuang, originating from the Qinghai-Tibet Plateau, were used as parents in this study. Haoyou 11 typically blooms ~20.63–26.21 days earlier than Dahuang under spring conditions and 33.83 days earlier under winter conditions. Haoyou 11 has been maintained for three generations through alternating self-mating and sib-mating, while Dahuang has been maintained over six generations by selfing. Reciprocal crosses were conducted between Haoyou 11 and Dahuang. F_1_ plants (Dahuang × Haoyou 11) were self-pollinated to generate F_2_ populations, which were used for the initial mapping of flowering time (Fig.  S5). To fine map the major QTL *qFTA06*, NILs were constructed using molecular MAS (Fig.  S5). F_1_ plants (Dahuang × Haoyou 11) were backcrossed with Dahuang to produce the BC_1_ segregating population. BC_1_ plants heterozygous for the target QTL region and predominantly homozygous in other genomic regions were backcrossed with Dahuang. This process was repeated for the BC_2_ and BC_3_ generations. BC_3_ plants heterozygous in the target QTL region and predominantly homozygous elsewhere were self-pollinated to produce the BC_3_F_2_ generation. Homozygous NILs harboring Haoyou 11 or Dahuang alleles within the *qFTA06* region were used for phenotypic evaluations, including flowering time and yield traits. Recombinants were identified from the BC_3_F_3_ segregating populations and self-pollinated to generate derived populations for progeny testing to further narrow the target interval.

### Field trial and trait evaluation

Flowering time was recorded as the number of days from seedling emergence to flowering. For individual plants, flowering time was defined as the days from seedling emergence to the opening of the first flower. For each field plot, flowering time was recorded as the number of days from when 75% of seedlings emerged to when 25% of plants in the plot flowered. Field trials for initial mapping and fine mapping were conducted during spring seasons from 2017 to 2021 in Xining City, Qinghai Province, China. Additional trials were conducted during autumn seasons from 2018 to 2020 in Yuanmou County, Yunnan Province, China. Plants were spaced 15 cm apart within rows and 30 cm between rows, with field management adhering to local agricultural practices.

To evaluate the potential negative effects of candidate flowering time genes on yield, agronomic traits of NILs were assessed. Early- and late-flowering NILs were sown in spring and harvested in autumn in Xining, Qinghai Province, China. Planting density and field management were consistent with QTL mapping protocols. A randomized block design was used to evaluate NIL phenotypes, with three replicates per environment and five lines per replicate. At the mature stage, 20–40 open-pollinated plants per NIL were harvested. Yield-related data, including plant height, yield per plant, seed number per silique, silique number per plant, and thousand seed weight, were calculated as mean ± standard deviations (SD) across three replicates for each environment.

### Statistical analysis

Descriptive statistical analyses, including means, variances, and standard deviations, were performed using IBM SPSS Statistics for Windows, version 29.0.2.0 (Armonk, NY: IBM Corp). Significance analysis was performed with *t-*test. Broad-sense heritability for flowering time was estimated using the formula: h${}_B^2$ = σ${}_g^2$/(σ${}_g^2$ + σ${}_e^2$), where h${}_B^2$ is broad-sense heritability; σ${}_g^2$ is genotypic variance; σ${}_e^2$ is error variance [60].

### GBS-based genetic linkage map construction and QTL detection

Genomic DNA was extracted from fresh leaves of 203 randomly selected individuals from the F_2_ population and both parents using the MiniBEST Plant Genomic DNA Extraction Kit (Takara, Dalian, China). Libraries were sequenced with 150 bp paired-end reads using the Illumina HiSeq™ platform (Novogene, Beijing, China).

Raw sequencing data underwent quality control (QC) using fastp software (version 0.23.0) to remove low-quality bases and adapter sequences. Clean reads from parents and offspring were mapped to the reference genome Chiifu v3.5 (http://brassicadb.cn/#/Download/) using Burrows-Wheeler Aligner (BWA) software (version 0.7.15-r1140) with the MEM algorithm [61]. The alignment results were transformed into BAM files with SAMtools software (version 1.3.1), and the reads were sorted [62]. GVCF files for each sample were generated using the HaplotypeCaller module in the GATK (version 3.7) software package. Single nucleotide polymorphisms (SNPs) and InDels variants were detected using the GenotypeGVCFs function in GATK [63]. ANNOVAR software (version 2016Feb1) was used to annotate variants based on the GFF3 files of the reference genome [64]. The genetic linkage map was constructed with the Kosambi mapping function.

QTL analysis was performed using the CIM method implemented in QTL Cartographer (version 1.17j). The QTL identification threshold was established by permutation testing (1000 iterations, *P* = 0.05). Additive effects (ADD) and dominance effects (DOM) of each flowering QTL were calculated, and QTL contributions were measured as the ratio of genetic variance to phenotypic variance. QTLs were named according to the convention in reference ^65^.

### Bulked-segregant analysis by sequencing (BSA-seq)

Extreme early- and late-flowering individuals (23 each) were selected from a population of 585 F_2_ plants to create early- and late-flowering gene pools. DNA was extracted, and libraries with 350-bp insertions were prepared and sequenced on the Illumina HiSeq™ platform PE150 by Novogene (Beijing, China). DNA from each line was pooled in equal ratios.

Raw data were processed using the same QC procedures as in GBS, and clean reads were aligned to Chiifu v3.5 genome. Variation calling was conducted with GATK, and results were saved in VCF files. The Δ(All-index) was calculated using QTLseqr (R package) software, and its distribution on each chromosome was plotted with a sliding window of 2 Mb. Simulations (1000 replicates) were performed for each bulk, and QTLs were identified at the 99% confidence level based on Δ(All-index) peaks.

### Development of molecular markers

Young leaf tissues were collected from seedlings for DNA extraction using a modified cetyltrimethylammonium bromide (CTAB) method, and the extracted genomic DNA was stored at −20°C [66]. SNP and InDel sites suitable for marker development were identified from resequencing data by screening specific sites flanking and within the candidate interval.

For the development of KASP markers, high-quality SNP/InDel sites with at least 75 bp of sequence on both sides were extracted and provided to Higenetec (Hunan, China) for primer synthesis and detection. KASP markers were primarily used to screen recombinant individuals in large populations, and genotyping results were analyzed with SNPviewer (http://www.lgcgroup.com).

Approximately 400 InDel markers at the specific locus within the candidate interval were validated for polymorphism in the parental lines, F_1_ generation, and extreme pools, leading to the identification of 60 clear-band markers for genotyping recombinants (Table S15). Selection criteria for InDel markers included primer lengths of 20–22 bp, a melting temperature (Tm) of 55°C, a GC content of 40%–60%, and amplicon sizes of 80–200 bp. PCR protocols and the 20 μl PCR system are detailed in Table S19. To enhance resolution, a 6% polyacrylamide gel electrophoresis (PAGE) was used to separate length polymorphisms. All primer sequences were verified for specificity in the NCBI database, and the sequences are listed in Table S15. InDel primers were synthesized by Genewiz Biotechnology Co., Ltd. (Suzhou, China).

### Fine mapping of *qFTA06*

Following the initial localization of the *qFTA06* locus on the chromosome, polymorphic markers and large secondary populations were developed to refine the target interval. In 2019 and 2020, two BC_3_F_3_ populations were cultivated, and flanking KASP markers were used to screen all recombinants in segregating populations. Recombinant plants were self-pollinated, and their progeny were genotyped using InDel markers within the interval. Recombinants were categorized based on their genotypes. In 2020, phenotypic and genotypic analyses of extreme phenotypic recombinants in the BC_3_F_3_ population preliminarily reduced the candidate interval. To validate and further narrow this interval, a recombinant-derived progeny testing strategy was employed in 2021. Recombinant plants were genotyped using newly developed InDel markers associated with *qFTA06*. Seventeen representative InDel markers were selected, classifying 277 recombinants into 12 types based on allele compositions and recombination breakpoints. Subsequently, 10–20 rows of recombinant offspring for each type were grown in experimental fields, and the flowering time phenotype of each plant was recorded. Offspring were genotyped into three categories: homozygous Haoyou 11, heterozygous, and homozygous Dahuang. Progeny testing was conducted by comparing the average flowering time phenotypes of the homozygous recombinants in each line. The candidate interval was narrowed down based on recombination breakpoints and statistical analyses of flowering time phenotypes.

### Candidate gene prediction and sequence analysis

Candidate genes were predicted based on the final mapping interval and the functional annotations of the reference genomes of *B. rapa* (Chiifu v3.5) and *B. napus* (ZS11 v0). Homologous gene information from *Arabidopsis* was retrieved via the TAIR database (www.arabidopsis.org). Using the full-length amino acid sequence of *BrCDF3* as a query, homologous proteins in other species were identified through the BLASTP program, employing an *E-value* threshold of 1.0 × 10^−5^. Sequence alignment was conducted using Clustal Omega, and a phylogenetic tree was constructed via the maximum likelihood method with 1000 bootstrap replicates in MEGA software (version 11.0.13). Gene structure prediction, protein sequence analysis, and conserved domain identification were performed using online tools such as EXPASY (https://www.expasy.org/), and the NCBI Conserved Domains Database (CDD) (https://www.ncbi.nlm.nih.gov/) were used for gene structure prediction, protein sequence analysis, and the identification of conserved domains.

Segment amplification primers (PS1, PS2, PS3), full-length primers, and CDS primers were designed using Primer 6.0 software, based on candidate gene sequences from the *B. rapa* Chiifu v3.5 genome. Genomic and coding sequences of the candidate gene were amplified from Haoyou 11, Dahuang, early-flowering NIL-E, and late-flowering NIL-L. Gene segments were amplified using the high-fidelity enzyme TaKaRa Ex Premier™ DNA Polymerase (Takara, Dalian, China) and subsequently cloned into the pMD19-T vector (Takara, Dalian, China). Positive clones were sequenced via Sanger sequencing at Sangon Biotech Co., Ltd. (Shanghai, China). Sequence assembly was conducted with SeqMan Pro software (version 11.1.0) from DNASTAR Navigator (version 11.1.0.54). The full-length gene was amplified using KOD FX High Success-rate DNA Polymerase (TOYOBO, Japan) and cloned into the pMDC43 vector. Positive clones were sequenced using KBseq at Sangon Biotech. CDS amplification was carried out with PrimeSTAR® Max DNA Polymerase (Takara *et al.*) and inserted into the pMD19-T. Sequences were verified by repeated sequencing and aligned using DNAMAN software (version 9.0.1.116). Primer sequences and PCR protocols for cloning are provided in Table S15.

### RNA extraction and qRT-PCR analysis

To analyze the relative expression levels of the five candidate genes, young leaves from the NILs were collected at 10 days post-emergence (two-leaf stage). The expression pattern of *BrCDF3* was examined in various tissues from NIL-E and NIL-L at different developmental stages. Five tissues (roots, stems, leaves, stem tips, and flowers) were collected for growth stage-specific and tissue-specific analyses. All samples were frozen in liquid nitrogen and stored at −80°C. Total RNA was extracted using the TaKaRa MiniBEST Plant RNA Extraction Kit (Takara, Dalian, China). First-strand complementary DNA (cDNA) was synthesized using the PrimeScript™ RT reagent Kit with gDNA Eraser (Takara, Dalian, China) following the manufacturer’s instructions.

The *BrACTIN7* (*BraA02g003220.3.5C*) and *BnACTIN7* (*BnaA02G0033600ZS*) gene were used as an internal control, with specific intron-spanning primers designed using Primer 6.0 software. qRT-PCR amplification was conducted on a LightCycler® 480 Instrument II (Roche Applied Science, Germany) using TB Green® Premix Ex Taq™ II (Takara, Dalian, China). Three biological replicates and three technical replicates were performed for each sample. Relative quantification was calculated using the 2^–ΔΔCt^ method. RNA samples were obtained from plants cultivated in a growth chamber with an average temperature of 23°C, 75% relative humidity, and a 16-h light/8-h dark photoperiod. PCR amplification conditions and primer sequences for expression analysis are detailed in Tables S15 and S20.

### Recombinant plasmids construction and plant transformation

The VIGS system was used to functionally characterize *CDF3*, following previously established protocols with modifications [67]. Phytoene desaturase (PDS) is a key enzyme in the carotenoid biosynthesis pathway. Silencing of this gene disrupts carotenoid synthesis, resulting in photobleaching of plant leaves. In this study, the *PDS* gene was employed as a reporter gene in the VIGS system. Gene-specific fragments of 245 bp from *BrCDF3* and 213 bp from *BrPDS* were inserted into the *XbaI* and *KpnI* restriction sites of the pTRV2 vector, respectively. Agrobacterium strain GV3101 containing pTRV1, pTRV2, pTRV2-*BrCDF3*, and pTRV2-*BrPDS* was cultured at 28°C until OD_600_ reached 0.8–1.0. The cultures were then resuspended to OD_600_ = 1.0 in infiltration buffer and incubated at room temperature for 4 h. Agrobacterium solutions with pTRV2-*BrCDF3*, pTRV2-*BrPDS*, and pTRV2 plasmids were mixed in equal proportions with the pTRV1 solution to serve as the experimental group, positive control, and negative control (pTRV2 and no infiltration treatment), respectively. Using an injection method, 200 μl of the mixture was infiltrated into the cotyledons of *B. rapa* seedlings until the entire leaf was saturated. For the first and second virus infiltrations, 10-day-old and 17-day-old NIL-L seedlings were used, respectively. Each treatment included 50 seedlings, with the remaining inoculum applied as root irrigation. The infiltrated seedlings were kept in darkness for 16 h. After 3 weeks, phenotypes were observed, and gene expression levels were assessed using qRT-PCR.

Given that the stable genetic transformation system for *B. rapa* (Dahuang) has not yet been established, *A. thaliana* was selected as the recipient species for ectopic expression of the *BrCDF3* gene derived from *B. rapa*. Specific primers, Comp-BrCDF3(E)-F/R, were designed to amplify the full-length *BrCDF3* sequence (5154 bp) from Haoyou 11, including the promoter, coding, and downstream regions. This full-length sequence was subsequently inserted into the pMDC43 vector using homologous recombination technology, and positive clones were submitted to Sangon Biotech for KBseq sequencing validation. Primer pairs 35S-BrCDF3(E)-F/R and 35S-BrCDF3(L)-F/R were then used to amplify the CDS of *BrCDF3* from Haoyou 11 and Dahuang, respectively. These CDS fragments were cloned into plant expression vectors to construct the recombinant plasmids 35S::BrCDF3(E) and 35S::BrCDF3(L). All three recombinant plasmids—pMDC43::BrCDF3(E), 35S::BrCDF3(E), and 35S::BrCDF3(L)—were individually transformed into *Agrobacterium tumefaciens* strain GV3101 and subsequently introduced into wild-type *A. thaliana* (Col-0) via Agrobacterium-mediated floral dip. Following kanamycin resistance screening and molecular identification using specific primers, homozygous transgenic lines of the T_3_ generation were successfully obtained. For phenotypic analysis of flowering time, four positive transgenic lines were selected from each experiment (complementation test and two overexpression experiments), with ~12 individual plants per line.

To further validate the role of *CDF3*, additional experiments were conducted. The CDS of *BnCDF3* from ZS11 was amplified by PCR using the primer sets 35S-*BnCDF3*-F/R. The amplified products were cloned into two *BsaI* restriction sites between the promoter and terminator of a modified pCAMBIA1300 vector via Golden Gate Assembly, generating the recombinant vector 35S::*BnCDF3*. Additionally, a CRISPR/Cas9 vector was constructed to simultaneously knock out two copies of the *CDF3* gene in ZS11 (*BnaA06G0175400ZS* and *BnaC03G0619100ZS*). The two sequences share 89.50% sequence identity, which poses a significant challenge in designing gene-specific sgRNAs. sgRNAs for CRISPR/Cas9 were designed using casTgtfinder (http://admin.biorun.com/admin/findcasit.asp), considering factors such as: (i) target specificity, (ii) location at the 5′ end of the CDS, (iii) avoidance of targets spanning introns, and (iv) a minimum GC content of 40%. Three targets were identified within the first exon of *CDF3*, upstream of the Dof conserved domain. These 20-bp protospacer adjacent motif sequences were cloned into the pBSbdcas9i vector containing the *AtU6–26* promoter via Golden Gate Assembly. Following Sanger sequencing, the Agrobacterium strain GV3101 was transformed with the overexpression and knockout vectors. ZS11, a semi-winter type of *B. napus*, was used as the transformation recipient via the Agrobacterium-mediated hypocotyl transformation method. An improved platform for high-throughput mutation detection (Hi-TOM 2.0) was used to sequence and analyze the mutations for each locus. The number of sequencing reads for each sample exceeds 10 000.

Transgenic plants were grown in an artificial climate chamber under a 16-h/23°C light and 8-h/23°C dark photoperiod with 75% relative humidity. Following germination, the seeds of *B. napus* undergo vernalization at 4°C for one month before being transplanted into the soil to monitor the dates of emergence and flowering. All transformants were screened for the presence of transgenes using PCR amplification. Positive transformants were confirmed, and target gene expression levels were evaluated by qRT-PCR. The primers, PCR amplification conditions, sequences of sgRNAs, and plant accessions used in these experiments are provided in Table S15.

### Subcellular localization

The coding region of *BrCDF3*, excluding the stop codon, was amplified from NILs using the primer pair Sub*BrCDF3*-EGFP (Table S15) and cloned in-frame into the expression vector pART-CAM-eGFP. A nuclear-localized marker gene (*AtH2B*) fused with mCherry and under the control of the 35S promoter was included as the reference. The empty pART-CAM-eGFP vector was used as a control. Recombinant plasmids (*BrCDF3*-eGFP co-infiltrated with *AtH2B*-mCherry) and the empty vector were transformed into *Arabidopsis* protoplasts. After 18 h at 23°C under low light conditions, fluorescence signals were observed using an Olympus FV1000 confocal laser scanning microscope. Green fluorescence was excited with a 488-nm argon-ion laser and observed within the 500–540 nm range. mCherry fusion protein fluorescence signals were excited with a 580-nm argon-ion laser and detected within the 570–670 nm range.

### Haplotype analysis of *BnA06CDF3* among natural populations

To investigate functional allelic variations and haplotype diversity of the *CDF3* gene in natural populations, we conducted a haplotype analysis of *BnA06CDF3* using Candihap software. For haplotype identification, we utilized resequencing data and flowering time phenotypes from 346 natural accessions of *B. napus*. Statistical significance was evaluated using *t*-test, and the results were visualized using the Chiplot online tool (https://www.chiplot.online/#).

## Supplementary Material

Web_Material_uhaf324

## Data Availability

Supporting data for this work are included in the manuscript and its supplementary files. The BSA and GBS datasets can be found in the NCBI database under SRA accession numbers PRJNA1214907 and PRJNA1214908.
