# Supplementary material for: Map-based cloning and functional characterization reveal CDF3 as the causal gene for the flowering time phenotype in Brassica rapa and Brassica napus
Source: Hortic Res. 2025 Nov 27;13(3):uhaf324. doi: 10.1093/hr/uhaf324 (PMC12966008; doi:10.1093/hr/uhaf324)
Supplement: Web_Material_uhaf324 [file web_material_uhaf324.zip › Supplementary Data Fig. S1-19.pdf]

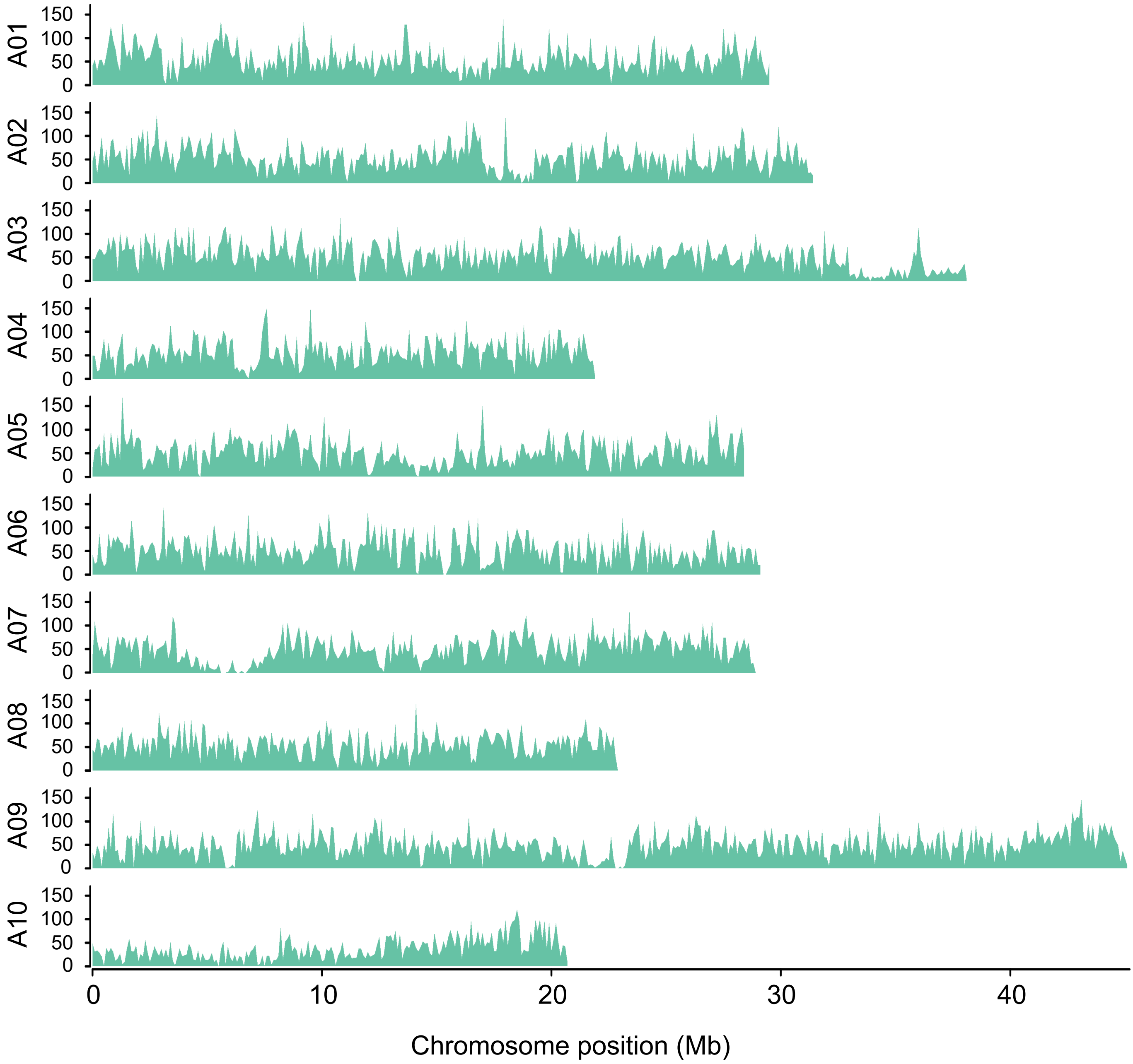

Supplementary Fig. 1 Genomic variation distribution between Haoyou 11 and Dahuang genomes based on simplified genome sequencing. A statistical analysis of genomic variant distribution was conducted using a 100 kb sliding window, with Chiifu v3.5 as the reference genome.

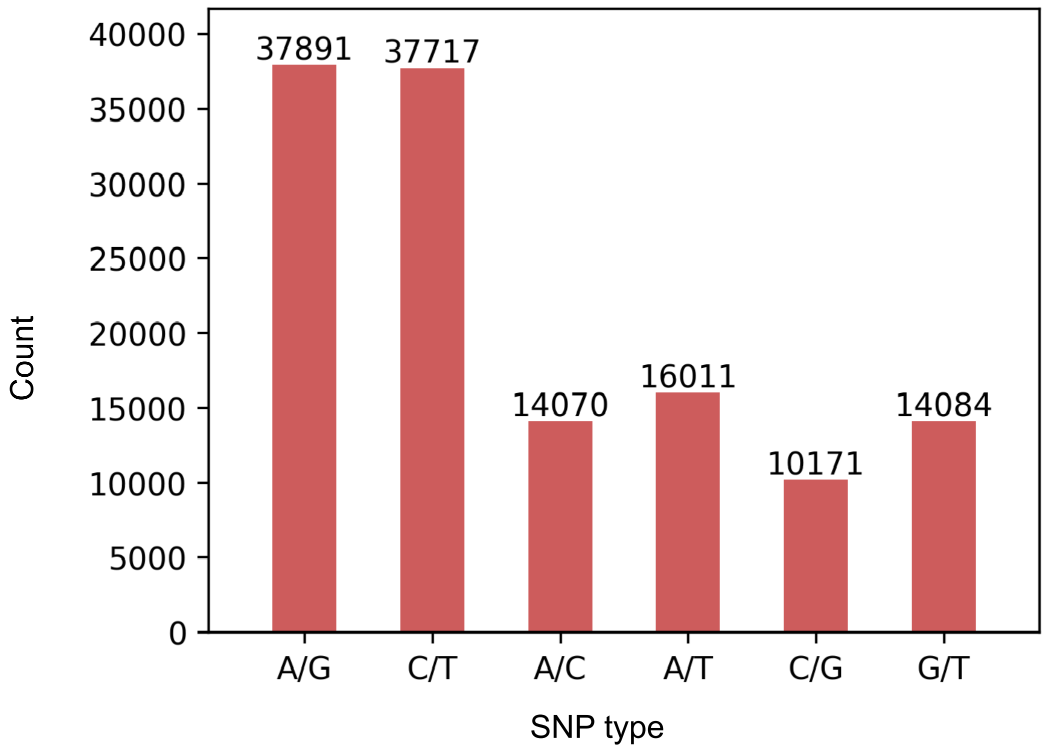

Supplementary Fig. 2 Details of Single Nucleotide Polymorphism (SNP) types based on simplified genome sequencing.

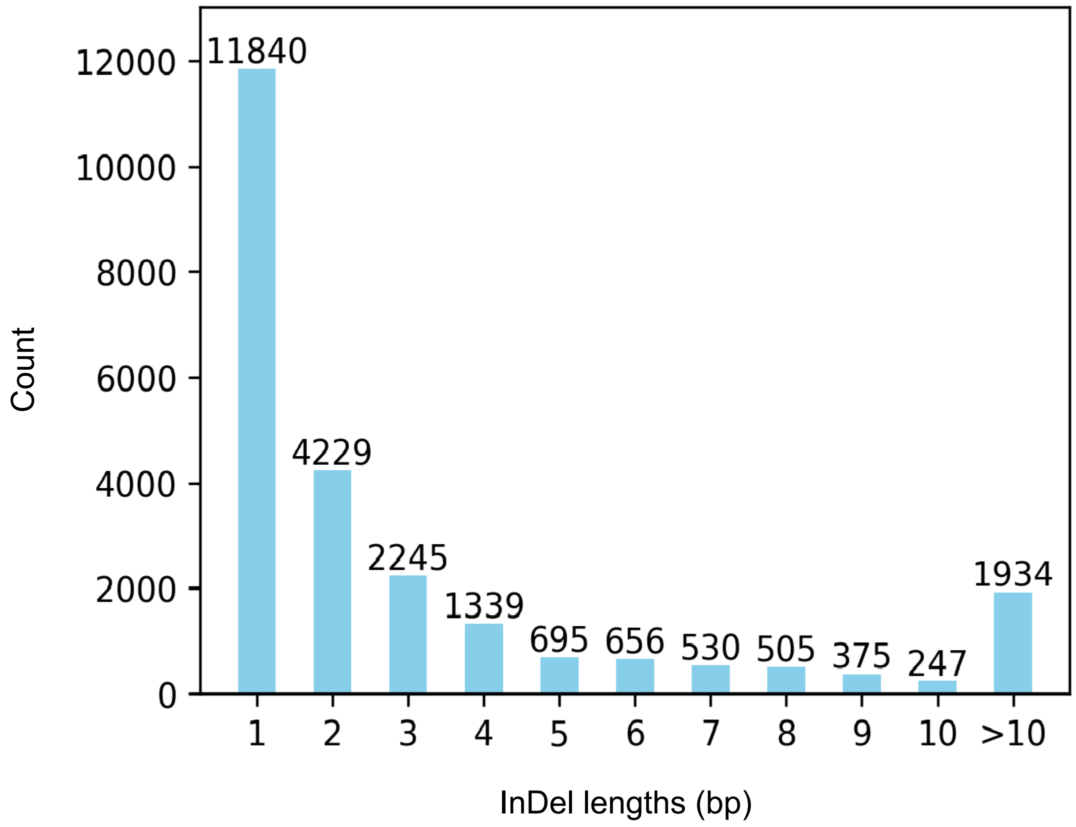

Supplementary Fig. 3 Details of Insertion and Deletion (InDel) lengths based on simplified genome sequencing.

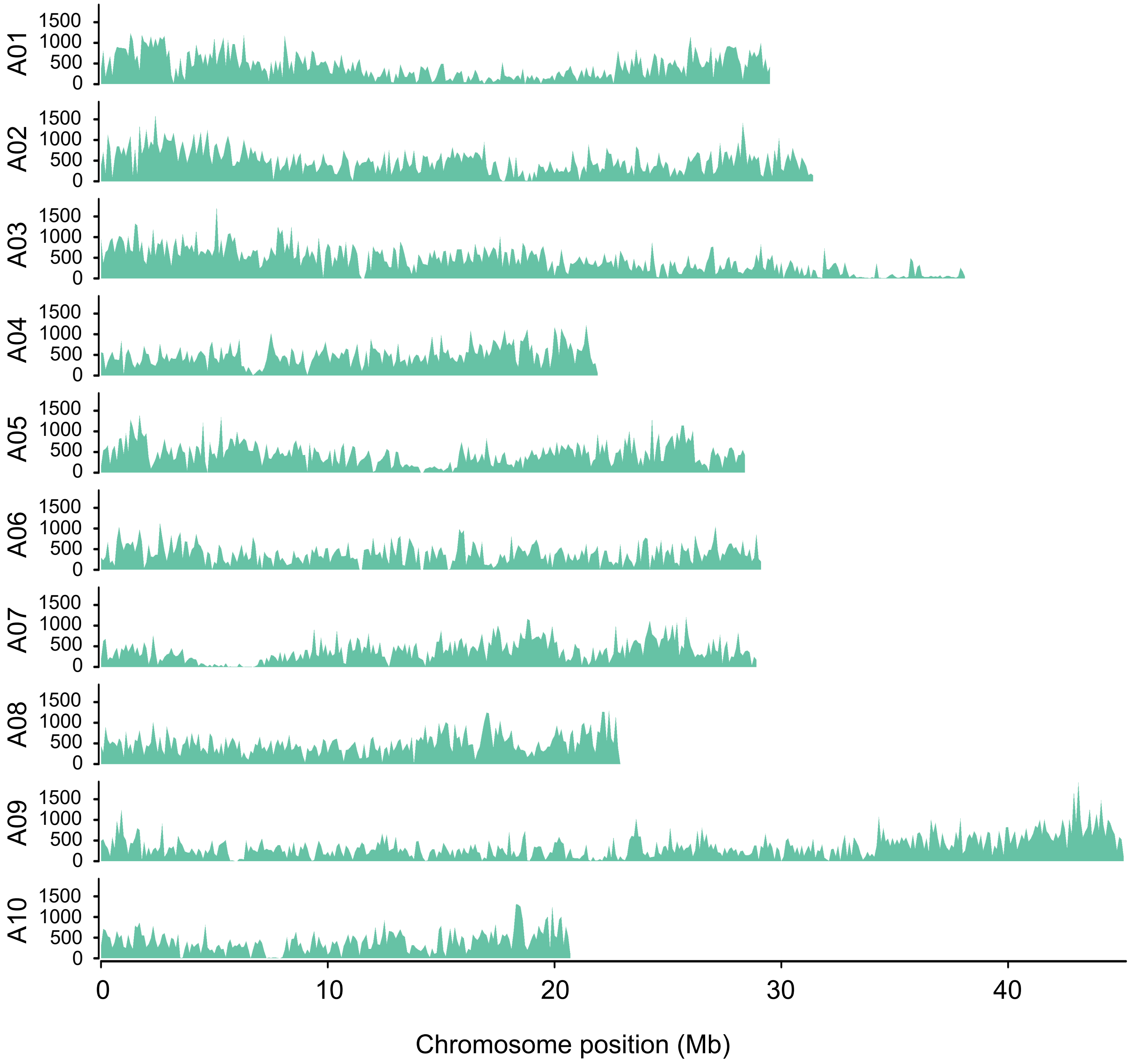

Supplementary Fig. 4 Genomic variation distribution between Haoyou 11 and Dahuang genomes based on BSA sequencing. A statistical analysis of genomic variant distribution was conducted using a 100 kb sliding window, with Chiifu v3.5 as the reference genome.

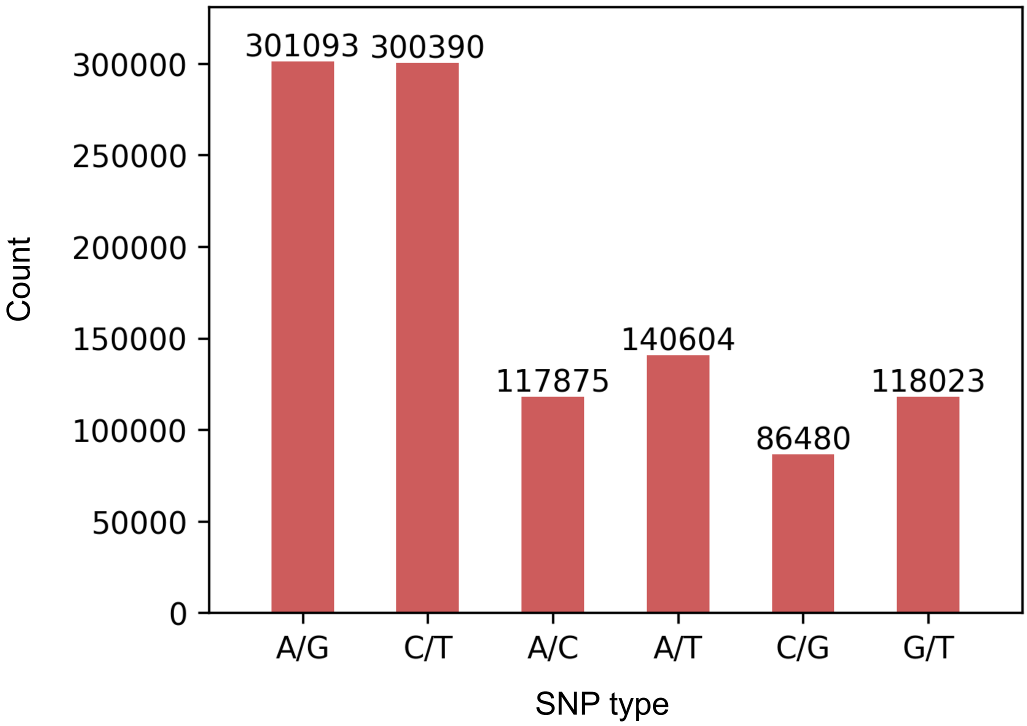

Supplementary Fig. 5 Details of Single Nucleotide Polymorphism (SNP) types based on BSA sequencing.

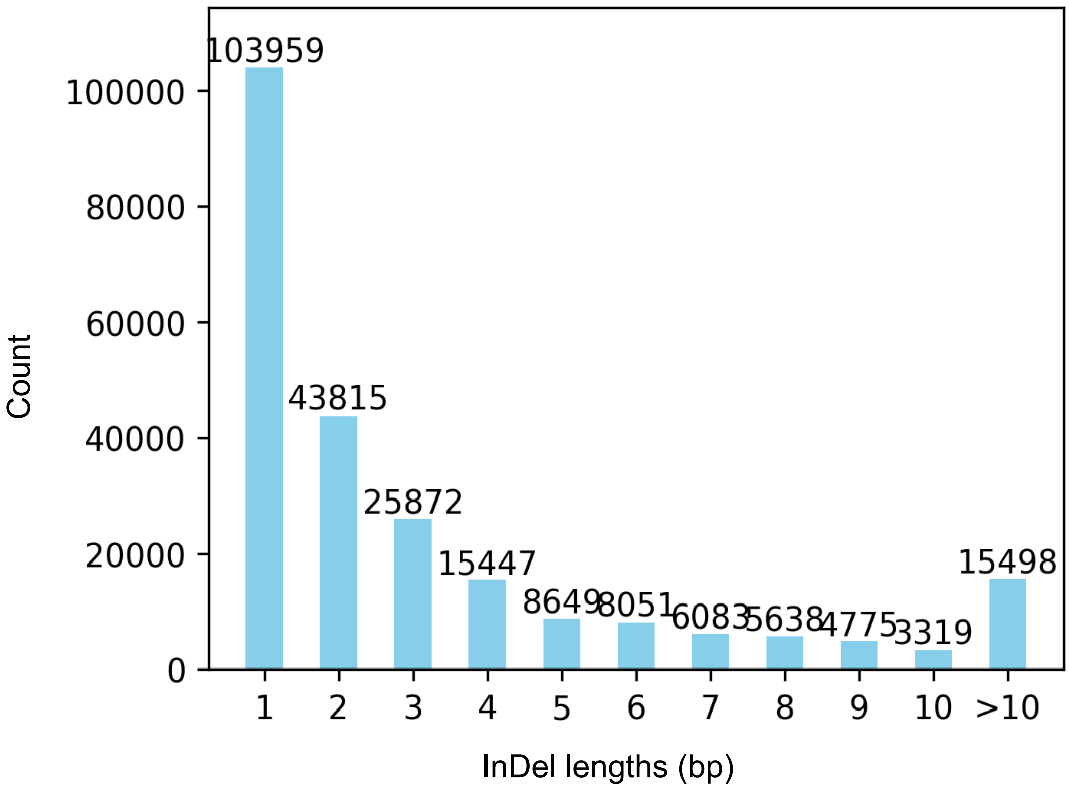

Supplementary Fig. 6 Details of Insertion and Deletion (InDel) lengths based on BSA sequencing.

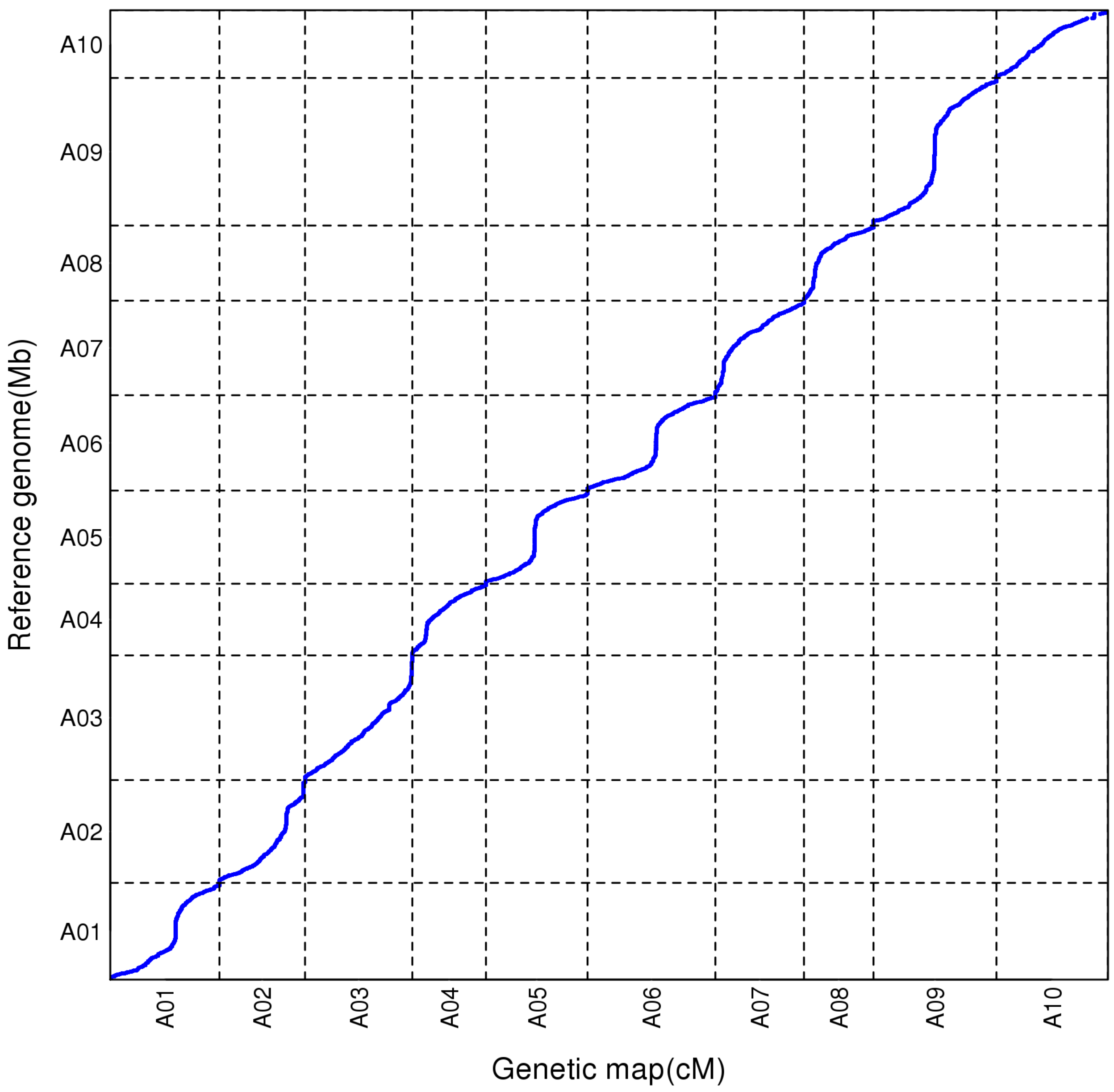

Supplementary Fig. 7 Collinearity analysis of the genetic and physical maps. The horizontal axis represents position on the genetic linkage map; the vertical axis indicates the corresponding physical location of the Chiifu reference genome.

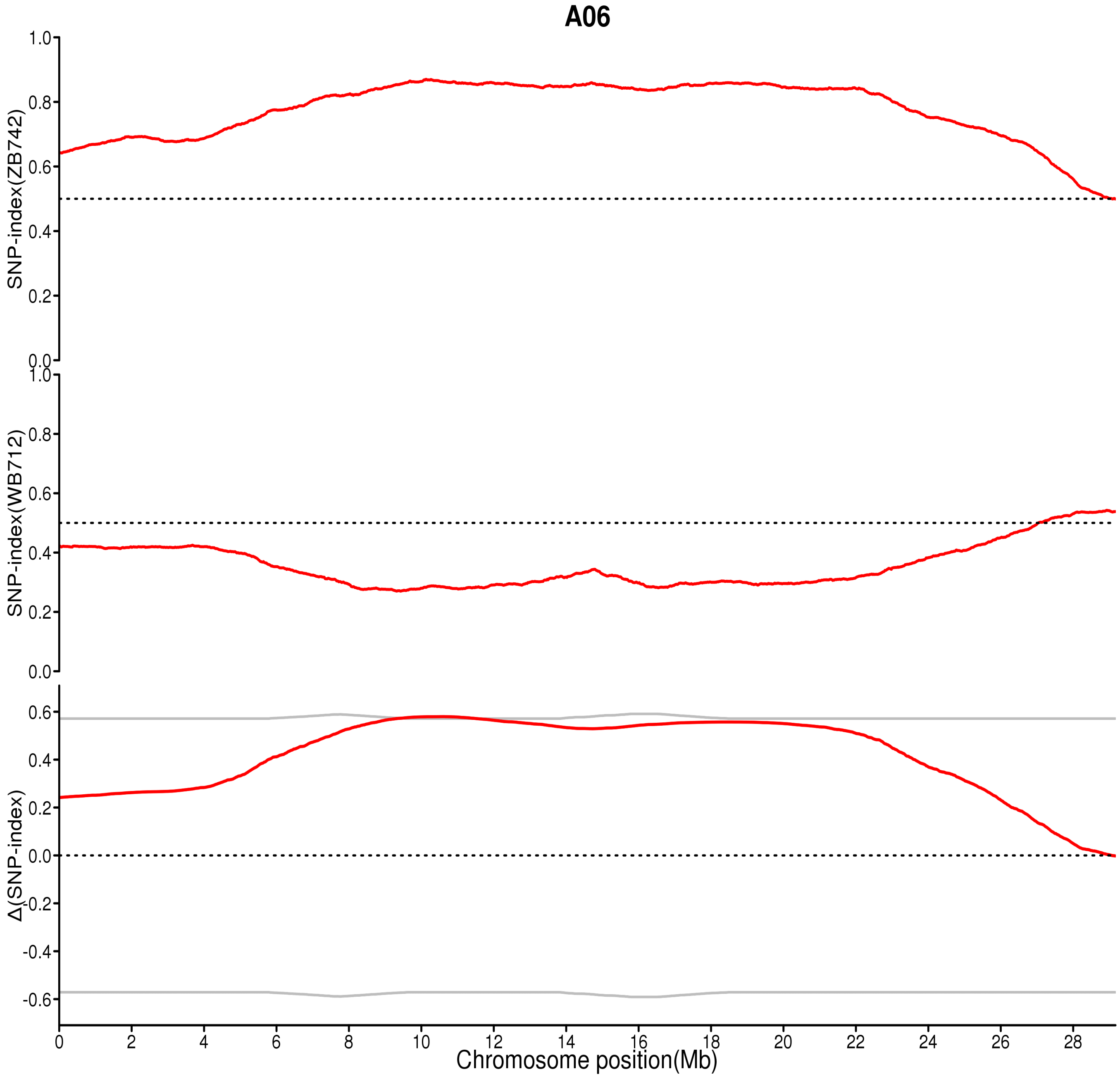

Supplementary Fig. 8 The distribution of Δ(SNP-index) values across chromosome A06. The red lines represent the average value of SNP-index/Δ(SNP-index) within each window; the X-axis represents chromosome length (Mb); the three Y-axes (from top to bottom) correspond to the SNP-index (E), the SNP-index (L), and the Δ(SNP-index). The gray line marks the 99% confidence interval threshold.

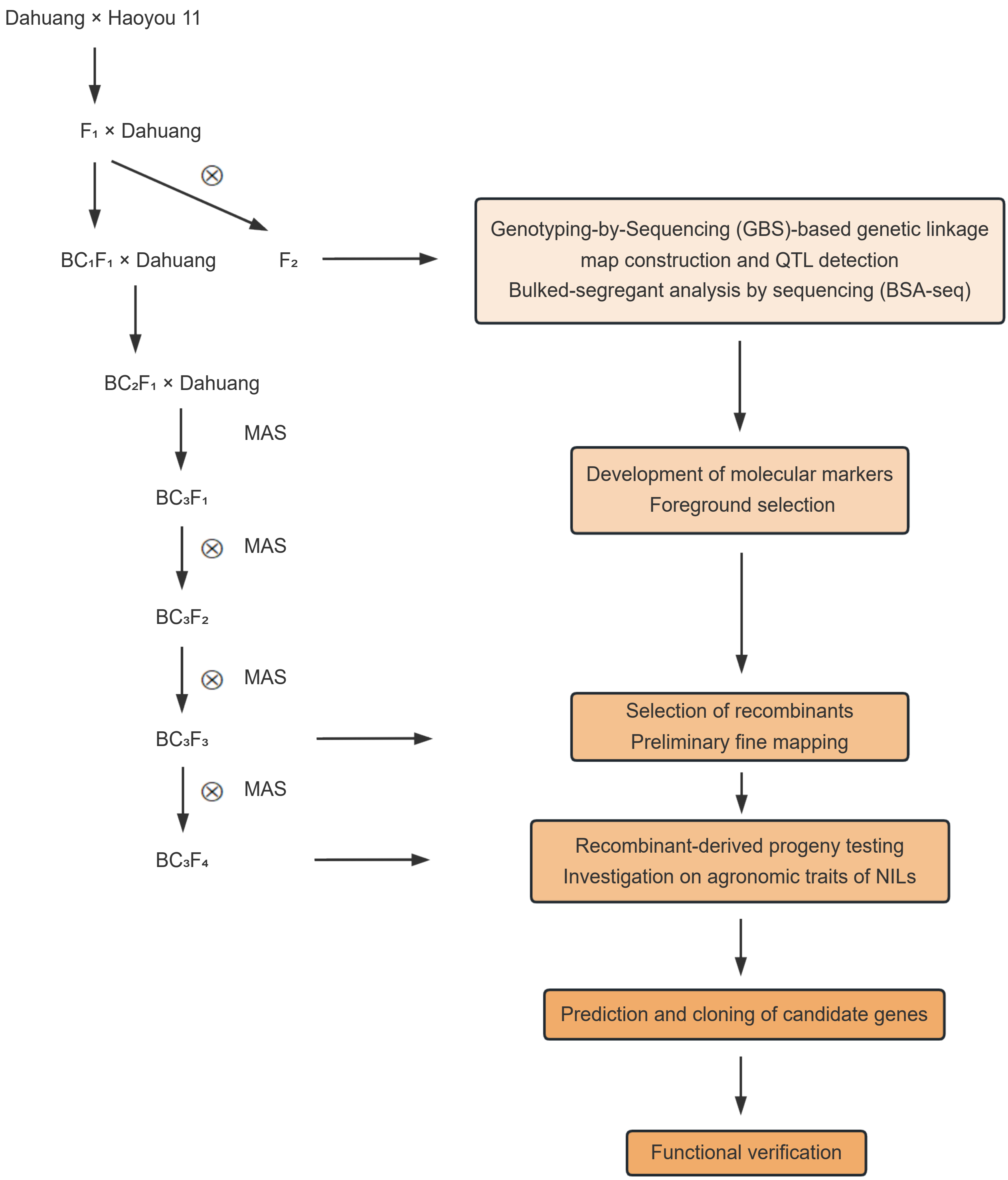

Supplementary Fig. 9 Breeding scheme used to construct the mapping population for flowering time analysis. x, crosses $\otimes$, self-pollination; MAS, molecular marker assisted selection.

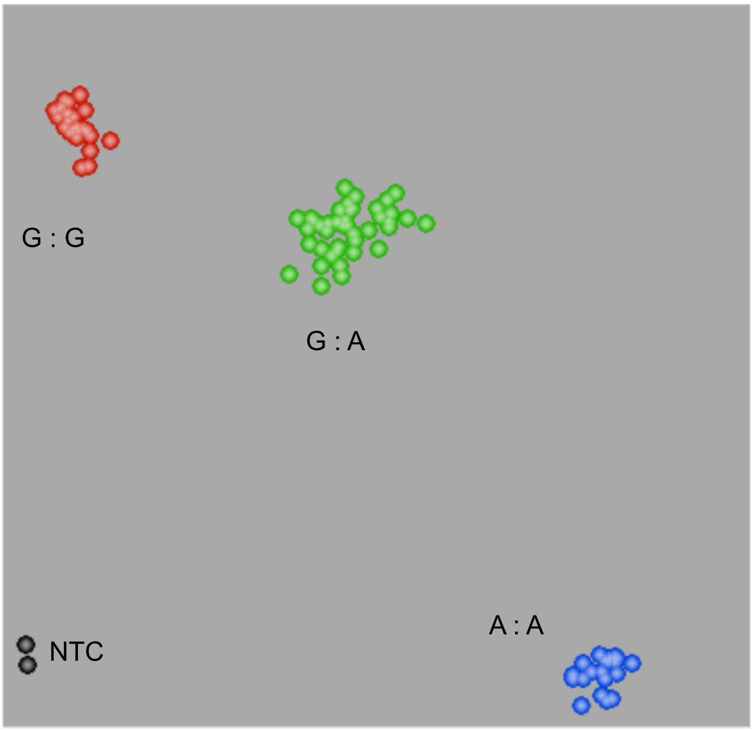

Supplementary Fig. 10 Genotyping data of KASP marker (A011624) using SNPviewer software. Red are homozygous for the allele reported with HEX, those marked blue are homozygous for the FAM allele and those marked green are heterozygous; black are Negative control or Non template control.

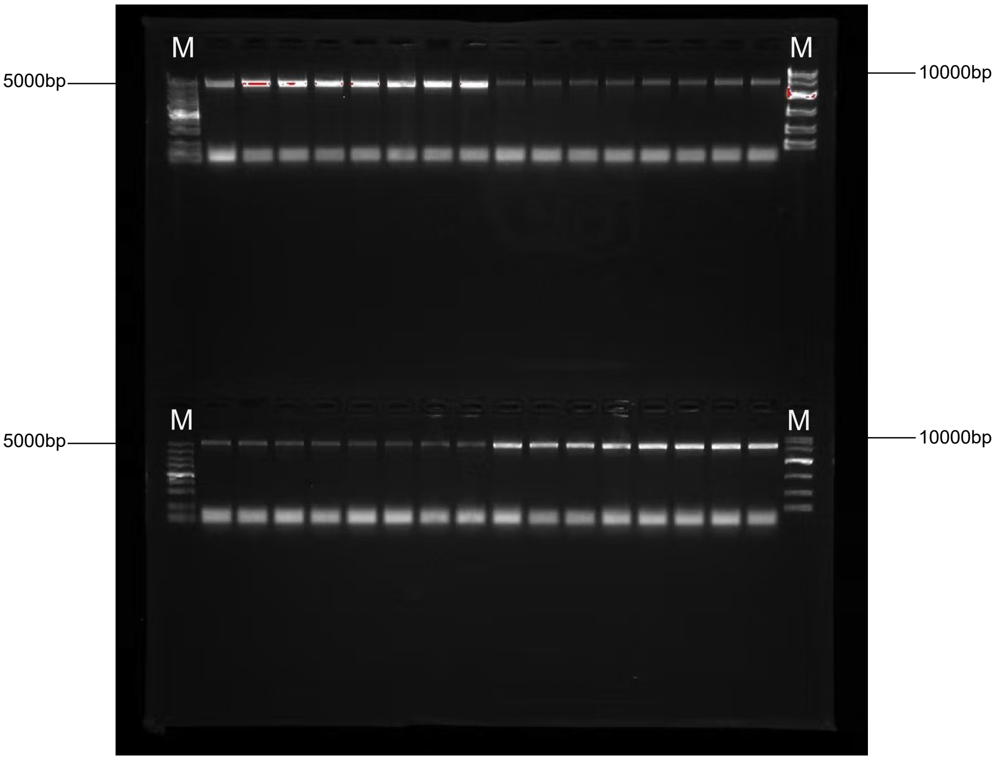

Supplementary Fig. 11 Amplification of full-length sequences. In the first row, the templates for 1-8 were Haoyou 11, whereas the templates for 9-16 were Dahuang. In the second row, the templates for 1-8 were NIL-E, and the templates for 9-16 were NIL-L. Both sides are markers.

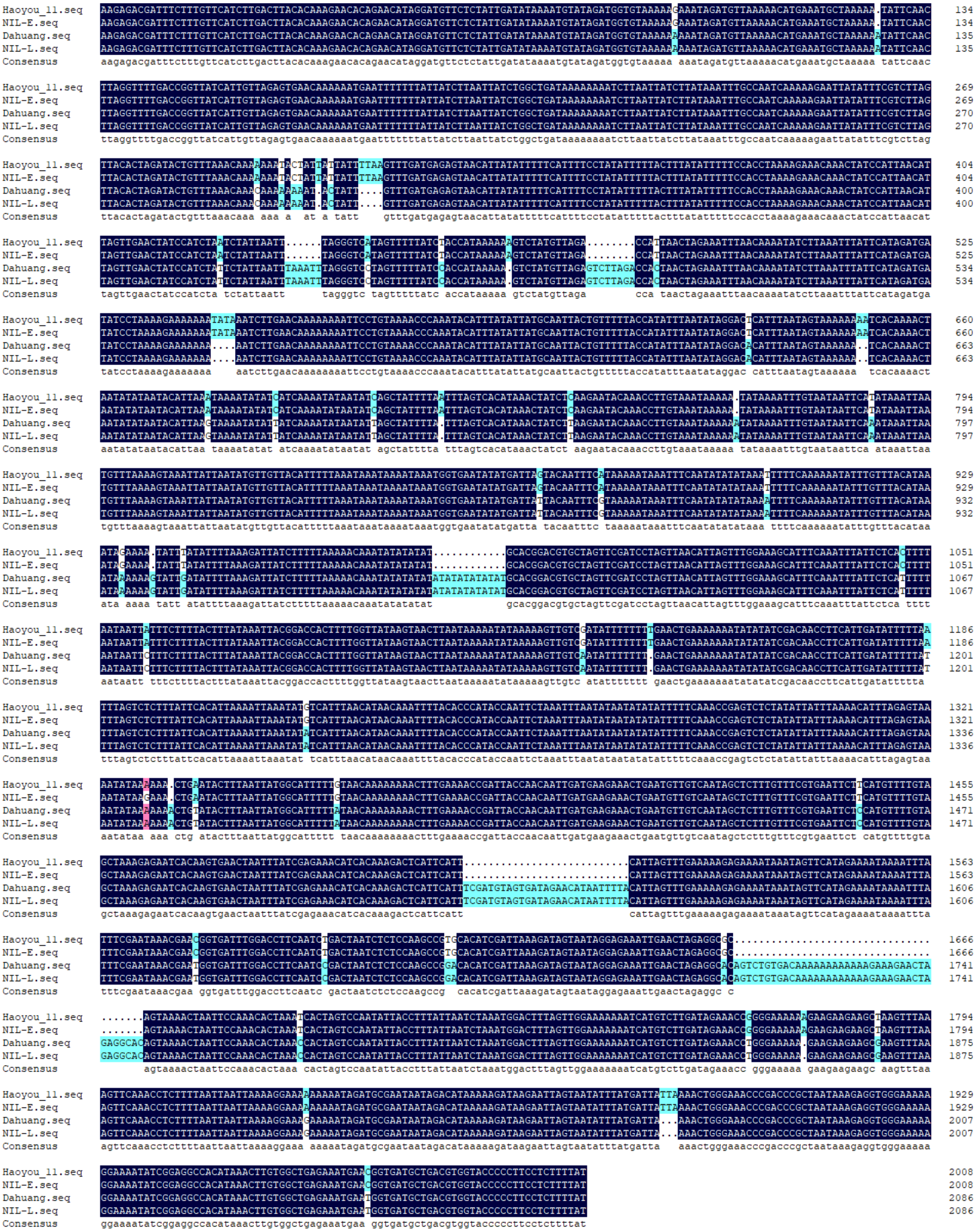

Supplementary Fig. 12 Comparison of the 2kb upstream regulatory regions between *BrCDF3-E* and *BrCDF3-L*.

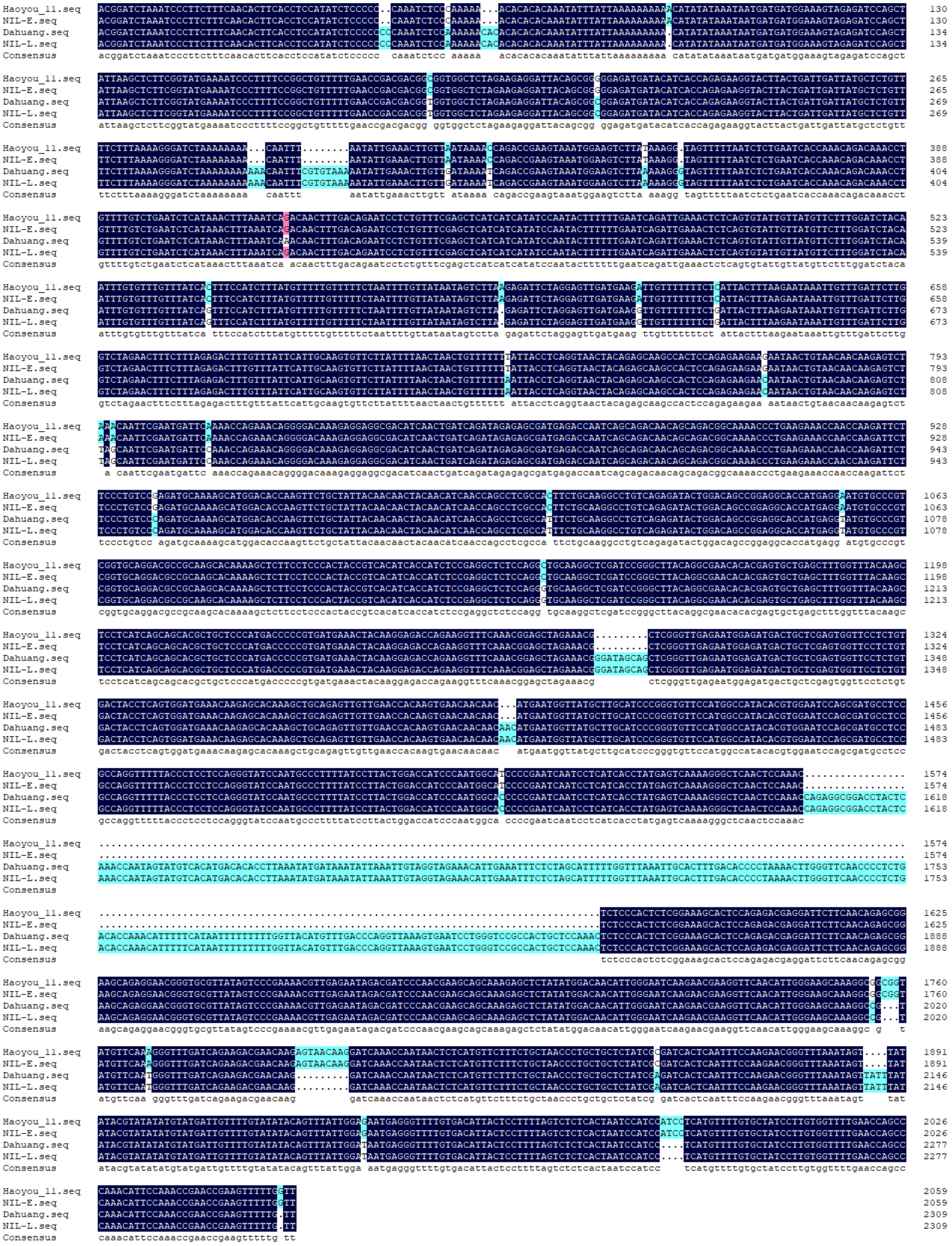

Supplementary Fig. 13 Comparison of the coding regions between *BrCDF3-E* and *BrCDF3-L*.

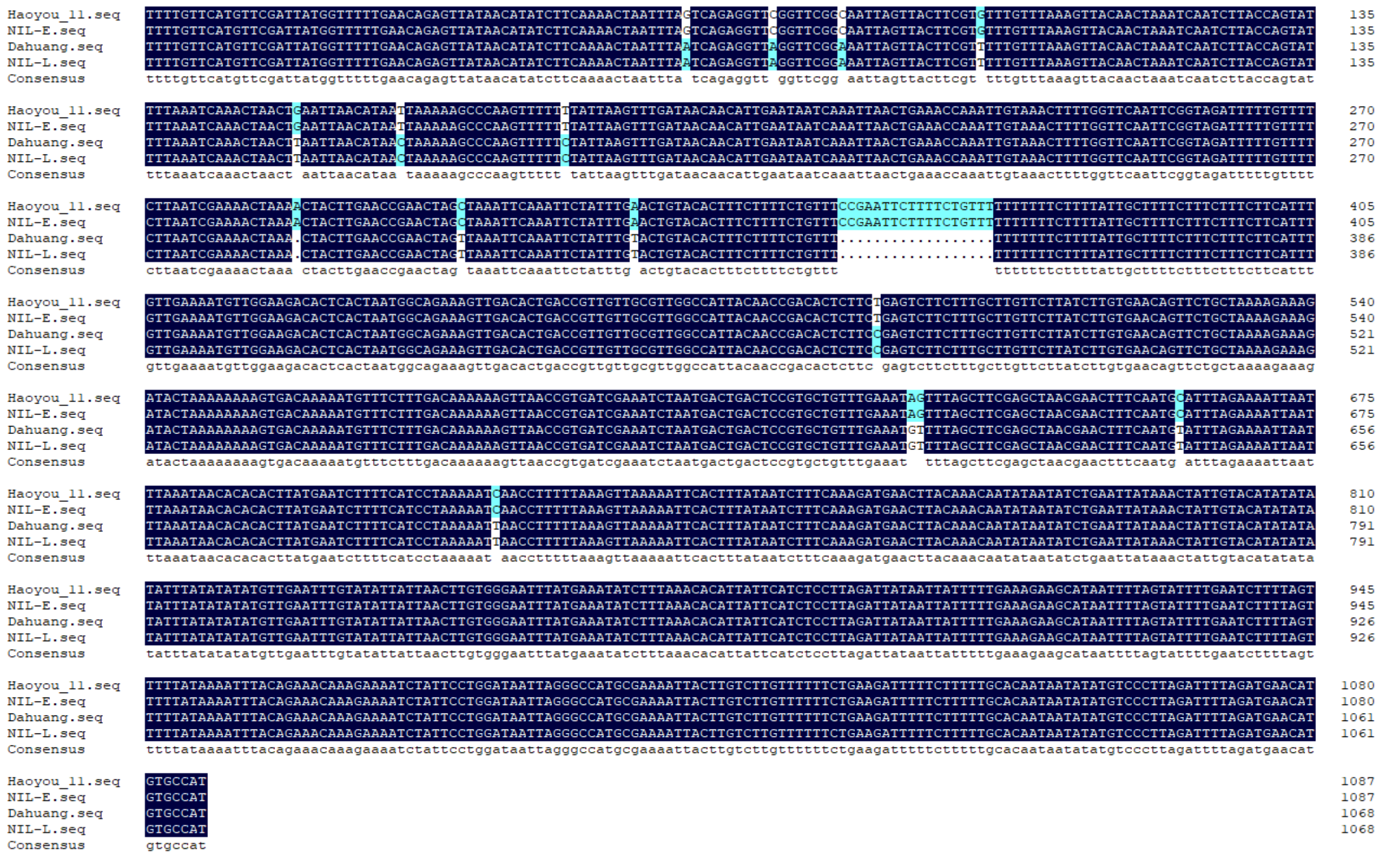

Supplementary Fig. 14 Comparison of the 1kb downstream regions between *BrCDF3-E* and *BrCDF3-L*.

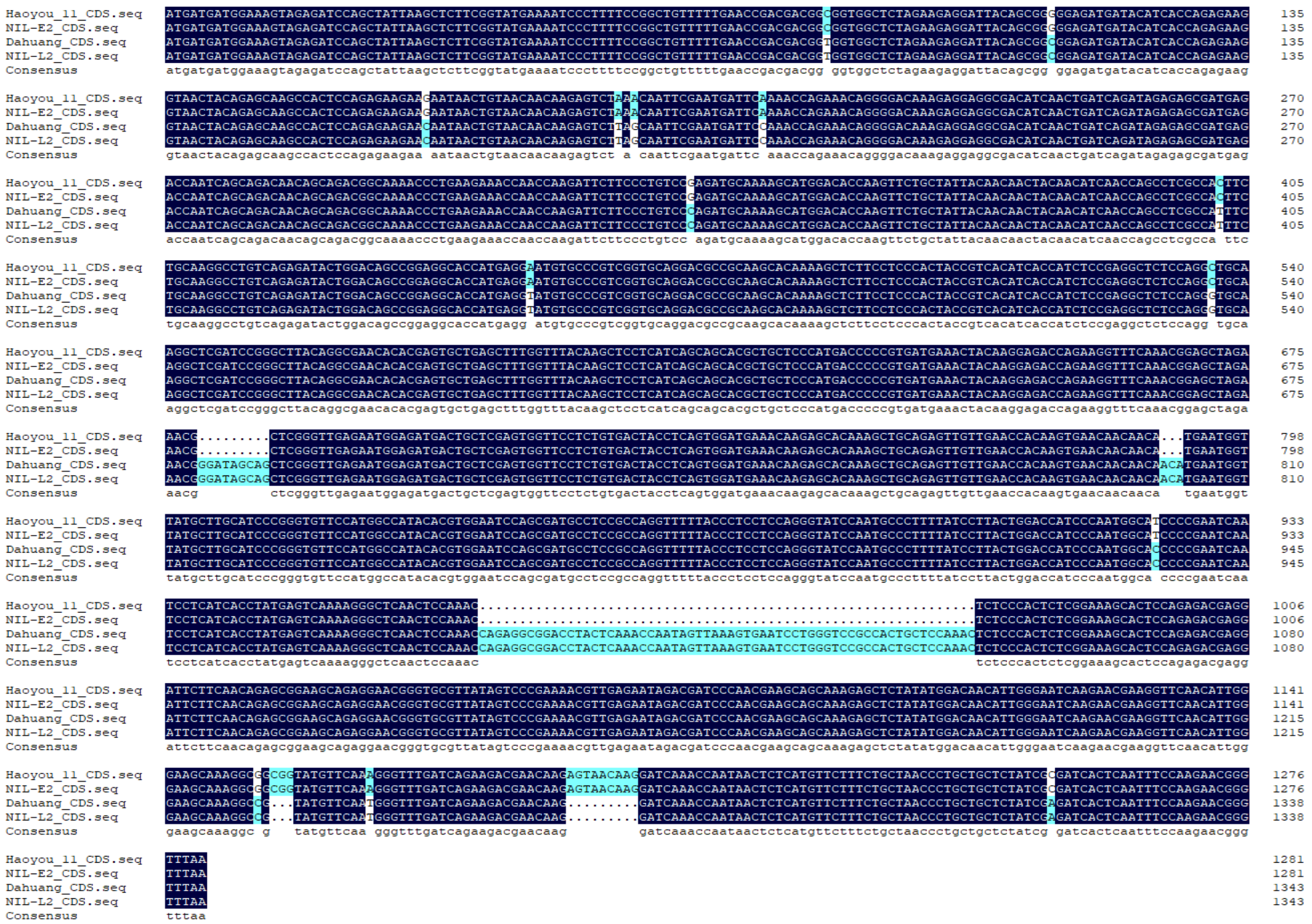

Supplementary Fig. 15 Comparison of the CDS between *BrCDF3-E* and *BrCDF3-L*.

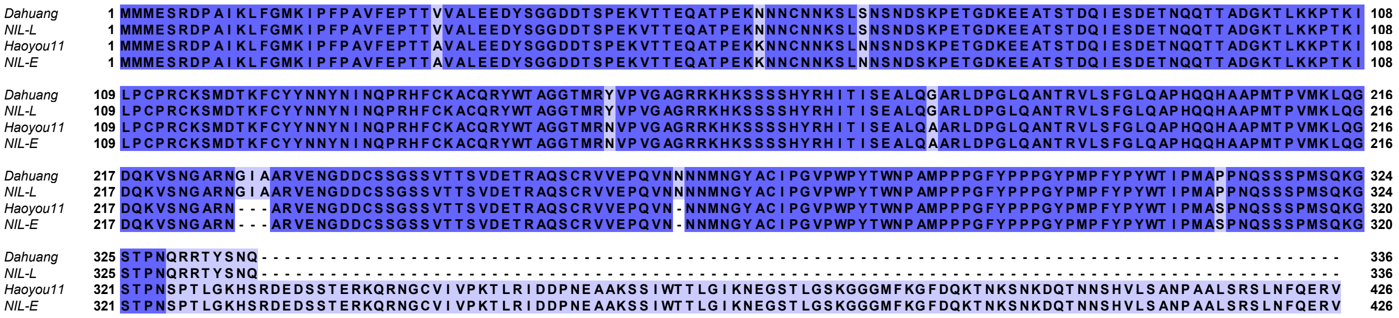

Supplementary Fig. 16 Comparison of amino acid sequences between BrCDF3-E and BrCDF3-L.

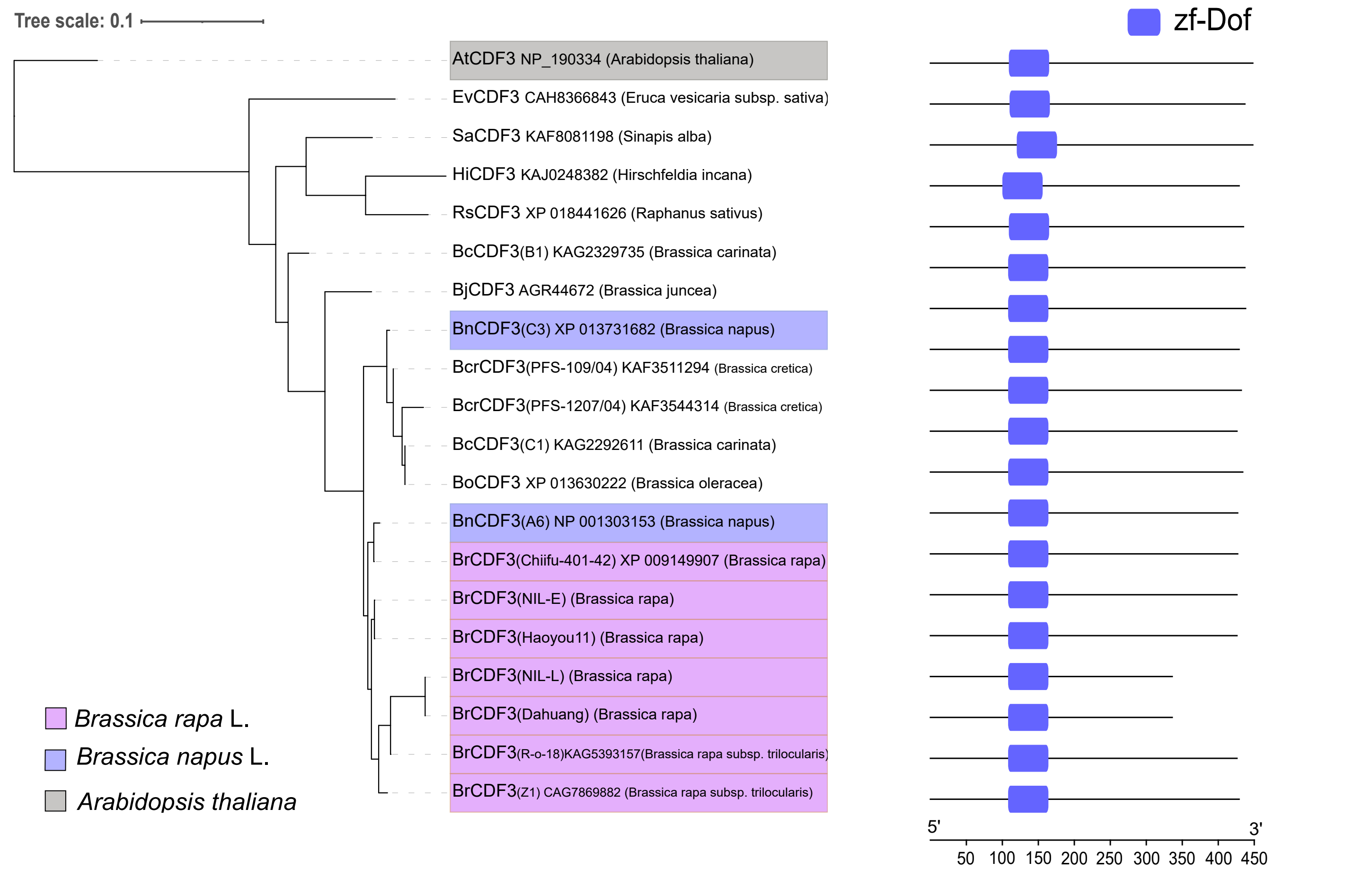

Supplementary Fig. 17 Phylogenetic tree and conserved domain analysis of BrCDF3 homologous proteins. The phylogenetic tree was constructed using the sequences of 16 homologous proteins of BrCDF3-E/L, presented on the left side.The conserved domains of these homologous proteins are displayed on the right side.

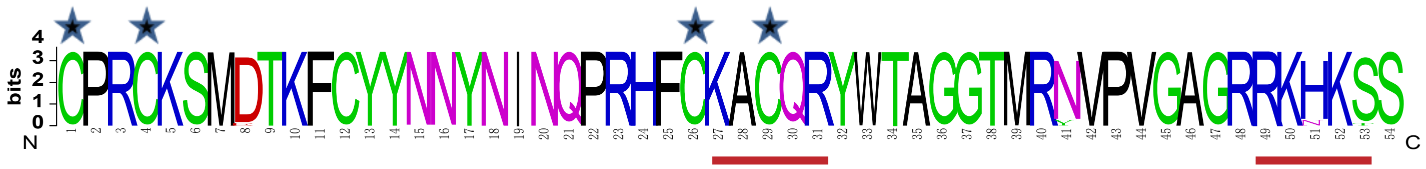

Supplementary Fig. 18 Conserved Dof domains in 20 homologous Dof proteins of BrCDF3. The sequence logos are based on alignments of 20 homologous Dof domains from BrCDF3. The bit score represents the information content for each position in the sequence. The core Dof domain contains 50 amino acid residues, located at sites 1-50. Asterisks denote the conserved four cysteine (Cys) residues within the Dof domain. Red bars denote the basic regions B1 and B2 of the bipartite NLS, as described by Krebs et al. (2010).

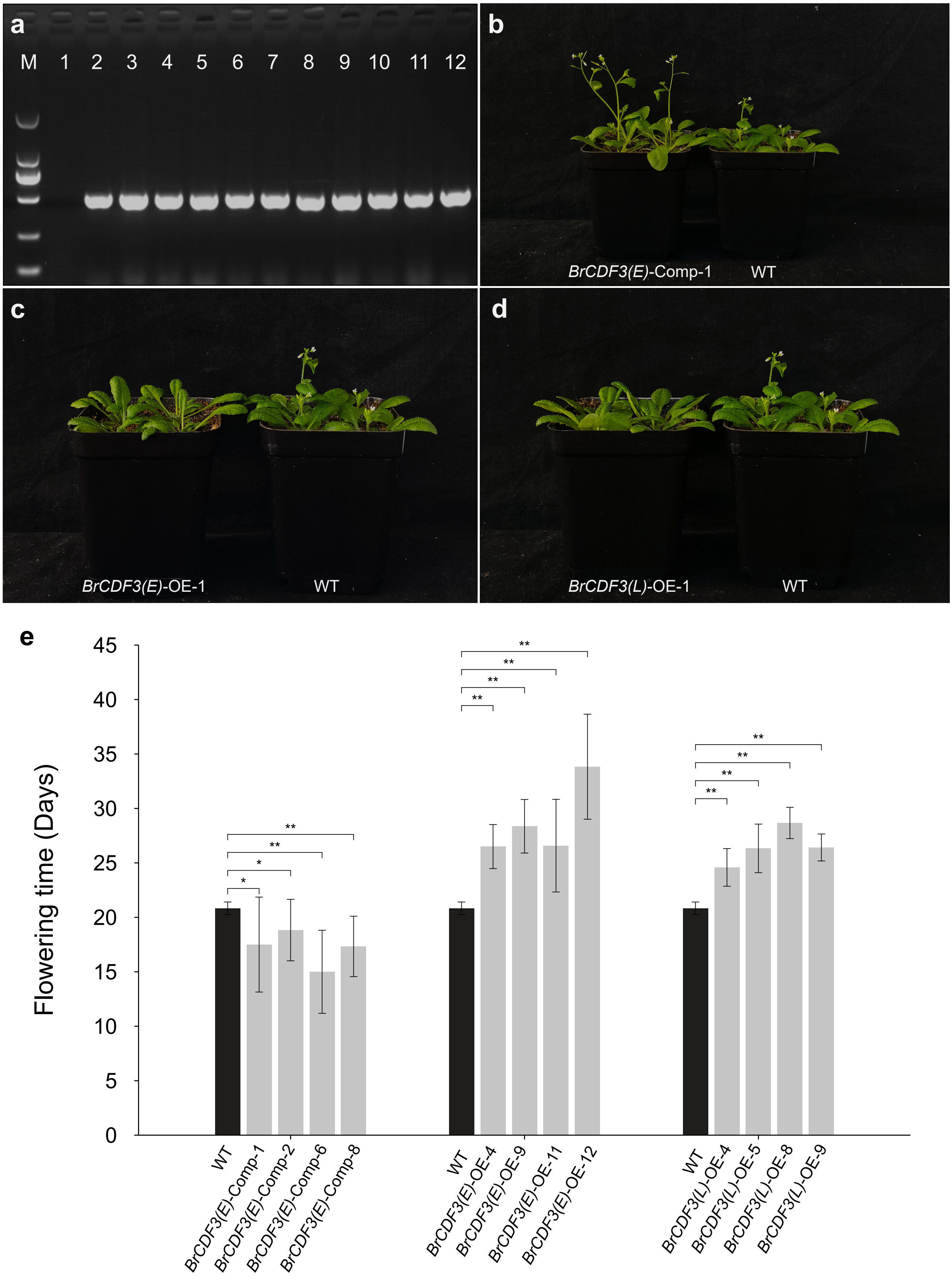

Supplementary Fig. 19 The stable transformation of the *BrCDF3* gene in *Arabidopsis thaliana*. a Identification of transgenic positive plants; b Phenotypes of *BrCDF3(E)*-Comp transgenic plants and control plants; c Phenotypes of *BrCDF3(E)*-OE transgenic plants and control plants; d Phenotypes of *BrCDF3(L)*-OE transgenic plants and control plants; e Flowering time in transgenic lines and wild-type plants in the T_3 generation. b–e employed the same wild-type Arabidopsis thaliana materials for comparative analysis. M represents the 2000 bp DNA ladder marker.
